# Neutral genetic drift can alter promiscuous protein functions, potentially aiding functional evolution

**DOI:** 10.1186/1745-6150-2-17

**Published:** 2007-06-28

**Authors:** Jesse D Bloom, Philip A Romero, Zhongyi Lu, Frances H Arnold

**Affiliations:** 1 Division of Chemistry and Chemical Engineering, California Institute of Technology, Pasadena, CA, 91125, USA

## Abstract

**Background:**

Many of the mutations accumulated by naturally evolving proteins are neutral in the sense that they do not significantly alter a protein's ability to perform its primary biological function. However, new protein functions evolve when selection begins to favor other, "promiscuous" functions that are incidental to a protein's original biological role. If mutations that are neutral with respect to a protein's primary biological function cause substantial changes in promiscuous functions, these mutations could enable future functional evolution.

**Results:**

Here we investigate this possibility experimentally by examining how cytochrome P450 enzymes that have evolved neutrally with respect to activity on a single substrate have changed in their abilities to catalyze reactions on five other substrates. We find that the enzymes have sometimes changed as much as four-fold in the promiscuous activities. The changes in promiscuous activities tend to increase with the number of mutations, and can be largely rationalized in terms of the chemical structures of the substrates. The activities on chemically similar substrates tend to change in a coordinated fashion, potentially providing a route for systematically predicting the change in one activity based on the measurement of several others.

**Conclusion:**

Our work suggests that initially neutral genetic drift can lead to substantial changes in protein functions that are not currently under selection, in effect poising the proteins to more readily undergo functional evolution should selection favor new functions in the future.

**Reviewers:**

This article was reviewed by Martijn Huynen, Fyodor Kondrashov, and Dan Tawfik (nominated by Christoph Adami).

## Open peer review

This article was reviewed by Martijn Huynen, Fyodor Kondrashov, and Dan Tawfik (nominated by Christoph Adami). For the full reviews, please go to the Reviewers' comments section.

## Background

Nature employs proteins for a vast range of tasks, and their capacity to evolve to perform diverse functions is one of the marvels of biology. Recently, it has become possible to reconstruct convincing scenarios for how new protein functions evolve. One of the most important conclusions of this work is that the initial steps may occur even before the new functions come under selection [1-6]. The reason is that in addition to their primary biological functions, most proteins are at least modestly effective at performing a range of other "promiscuous" functions [1,2,7-10]. In laboratory experiments, selection can rapidly increase these promiscuous functions, often without much immediate cost to a protein's original function [2]. In a particularly compelling set of experiments, Tawfik and coworkers have shown that selection for a promiscuous activity likely explains the origin and evolution of a bacterial enzyme that hydrolyzes a synthetic compound only recently introduced into the environment [2,11,12]. Mounting evidence therefore supports the idea that new protein functions evolve when selection favors mutations that increase an existing weak promiscuous function.

But for as long as 50 years, since Linus Pauling and Emile Zuckerkandl published their seminal analysis of molecular change in proteins [13], it has been clear that only a fraction of the mutations that accumulate in most naturally evolving proteins are driven by selection for an entirely new function. Instead, many of the mutations responsible for natural sequence divergence do not change a protein's primary biological function, but rather are due to either neutral genetic drift [14] or pressure for a subtle recalibration of protein properties unrelated to the acquisition of an entirely new function [15]. However, mutations that accumulate under the constraint that they not interfere with a protein's primary function could still substantially alter other, promiscuous functions. Such alterations could then aid in the subsequent evolution of new functions.

Here we have experimentally investigated this possibility using a set of enzymes that have undergone genetic drift that is neutral with respect to a well-defined laboratory selection criterion for enzymatic activity on a single substrate [16]. We have examined how these enzymes have changed in their promiscuous activities on five other substrates. As described below, we find that the enzymes have often undergone substantial changes in their promiscuous activities, suggesting that neutral genetic drift could play an important role in enabling future functional evolution.

## Results and Discussion

### A set of neutrally evolved cytochrome P450 enzymes

We focused our analysis on cytochrome P450 proteins. P450s are excellent examples of enzymes that can evolve to catalyze new reactions, since they are involved in a wide range of important functions such as drug metabolism and steroid biosynthesis [17,18]. We worked with P450 BM3, a cytosolic bacterial enzyme that can perform a variety of hydroxylation and epoxidation reactions, and is thought to have a natural function that includes the hydroxylation of fatty acids [19,20]. We have previously described a set of P450 BM3 heme domain variants that were created by laboratory neutral evolution from a common parent sequence [16]. Here we briefly recap the procedure used to create these P450s in order to explain their origin and why they can properly be viewed as the product of neutral genetic drift, albeit under a selection criterion that represents a severe simplification of the pressures likely faced by P450s evolving in nature.

The essential difference between neutral genetic drift and adaptive evolution is that in the former case mutations that have no substantial effect on fitness spread stochastically in a population, while in the latter case mutations spread because they are beneficial and so favored by selection. Of course, it may be difficult to discern whether a specific mutation in a natural population has spread neutrally or due to favorable selection. But in the laboratory it is possible to define an arbitrary selection criterion to ensure that all mutations spread due to neutral genetic drift. Specifically, we imposed the requirement that the P450s had to hydroxylate the substrate 12-*p*-nitrophenoxydodecanoic acid (12-pNCA) with an activity exceeding a specific threshold [16]. All mutant P450s were therefore straightforwardly classified as either functional (if they exceeded the threshold) or nonfunctional (if they did not). We readily acknowledge that the situation is almost surely more complicated for natural P450 BM3 enzymes, which are probably selected for several different enzymatic functions (the natural substrates of these enzymes are not definitively known [19,20]). In addition, we make no attempt to define how changes in a P450's enzymatic functions might map to changes in the overall fitness of its host organism, since there is not sufficient data on the metabolic and ecological role of P450 BM3 to convincingly reconstruct such a mapping. Instead, our selection criterion is simply an abstraction of the evolutionary requirement that an enzyme's primary activity must exceed some critical level in order to allow its host organism to robustly survive and reproduce. To implement laboratory neutral evolution using this selection criterion, we began with a single parent P450 BM3 heme domain variant (called R1-11) and used error-prone PCR to create random mutants of this parent [16]. Mutants that failed to yield sufficient active protein to hydroxylate at least 75% of the 12-pNCA of the R1-11 parent when expressed in *Escherichia coli *were immediately eliminated, while all other mutants were carried over to the next generation with equal probability. Any mutations that spread among the offspring sequences were therefore by definition due to neutral genetic drift, since there was no opportunity for any functional mutant to be favored over any other.

Throughout the remainder of this work, we will refer to mutations that spread by neutral genetic drift during our experiments as "neutral" – the reader should keep in mind that these mutations might not be neutral in all other evolutionary scenarios. In addition, in many natural settings a mutation may be slightly deleterious or slightly advantageous. A more nuanced description would therefore characterize a full fitness landscape containing different gradations of fitness [21,22], with evolution moving along ridges of high fitness [23]. Because the binary functional/nonfunctional classification made in our experiments ignores such gradations, these ridges in the fitness landscape are reduced to a neutral network, with all mutants either on the network (if they are functional) or off the network (if they are nonfunctional). We also emphasize that the fact that the mutations spread due to neutral genetic drift does not mean that they have no effect on the protein's properties. Indeed, one of the growing realizations about protein evolution is that mutations that spread by neutral genetic drift may still have an impact on future evolution [24,25]. One mechanism for this impact is that neutral genetic drift can change a protein's stability and so alter its tolerance to future mutations [26-28]. As will be demonstrated below, another mechanism is that neutral genetic drift can alter a protein's promiscuous functions.

**Figure 1:**
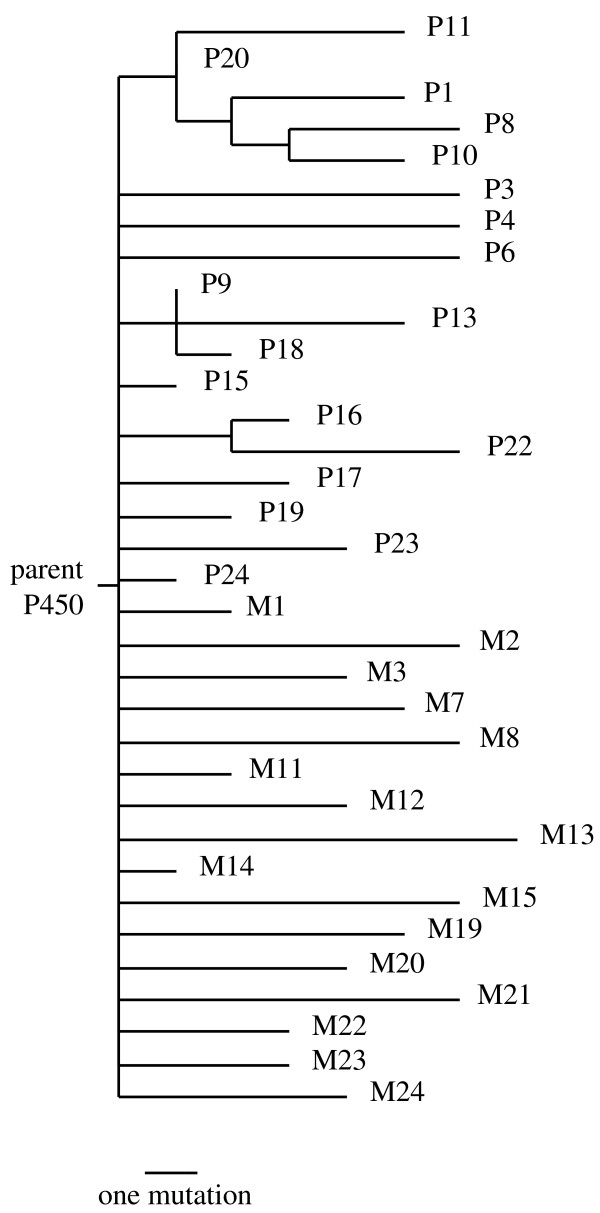
**Phylogenetic tree of the neutrally evolved P450s**. The tree shows the relationship among the 34 neutrally evolved P450 variants examined in this study. All of the P450s neutrally evolved from the same R1-11 parent P450. The horizontal lengths of the branches are proportional to the number of nonsynonymous mutations, as indicated by the scale bar. The vertical arrangement of the branches is arbitrary.

As described previously [16], the end result of the neutral evolution was 44 different P450 variants, each of which satisfied the selection criterion for activity on 12-pNCA (these are the combined final sequences from the monomorphic and polymorphic populations in [16]). For the current study, we analyzed the promiscuous activities of 34 of these neutrally evolved P450 variants. The sequence diversity of these P450s is shown in the phylogenetic tree of Figure 1 and the full genotypes are given in Additional file 1; they have accumulated an average of four nonsynonymous mutations each. Most of the mutations in the P450 variants are unique – out of a total of 105 different amino acid substitutions, only twelve occur in more than variant (Additional file 1).

### Activities of the neutrally evolved P450 enzymes

**Figure 2:**
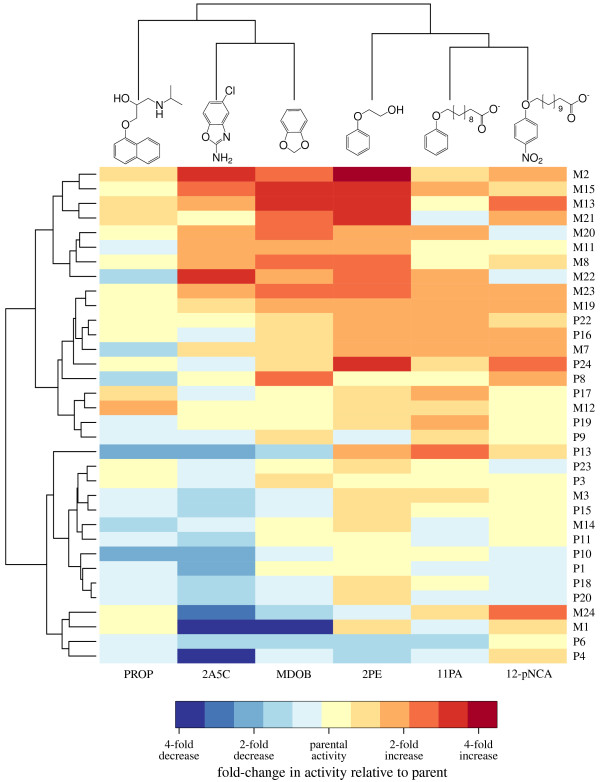
**Activities of the neutrally evolved P450s on the six substrates**. The heat map shows the fold change in activity of all 34 neutrally evolved P450 variant on all six substrates. Each row shows the data for a different P450 variant, while each column shows the activity on a different substrate. The fold change in activity is the ratio of the variant's activity to that of the neutral evolution parent. Both the substrates and the P450 variants are hierarchically clustered according to the activity profiles, as shown by the dendrograms at top and left. Substrate abbreviations are the same as those used in the text: PROP – propranolol, 2A5C – 2-amino-5-chlorobenzoxazole, MDOB – 1,2-methylenedioxybenzene, 2PE – 2-phenoxyethanol, 11PA – 11-phenoxyundecanoic acid. The standard errors for the changes in activity displayed in the heat map tend to be much smaller than the changes themselves; these errors are shown explicitly in Figure 3.

All of the P450 variants had evolved under selection solely for their ability to hydroxylate 12-pNCA. We examined their promiscuous hydroxylation activities on the five other substrates shown at the top of Figure 2. Two of these promiscuous substrates, propranolol (PROP) and 2-amino-5-chlorobenzoxazole (2A5C, also known as zoxazolamine), are drugs that are metabolized by human P450s [29,30]. The other three promiscuous substrates, 11-phenoxyundecanoic acid (11PA), 2-phenoxyethanol (2PE), and 1,2-methylenedioxybenzene (MDOB), are organic compounds of increasing structural dissimilarity to 12-pNCA. The parent P450 possessed at least some hydroxylation activity on all of these substrates (throughout the remainder of this work, "activity" refers to total substrate turnovers per enzyme).

**Figure 3:**
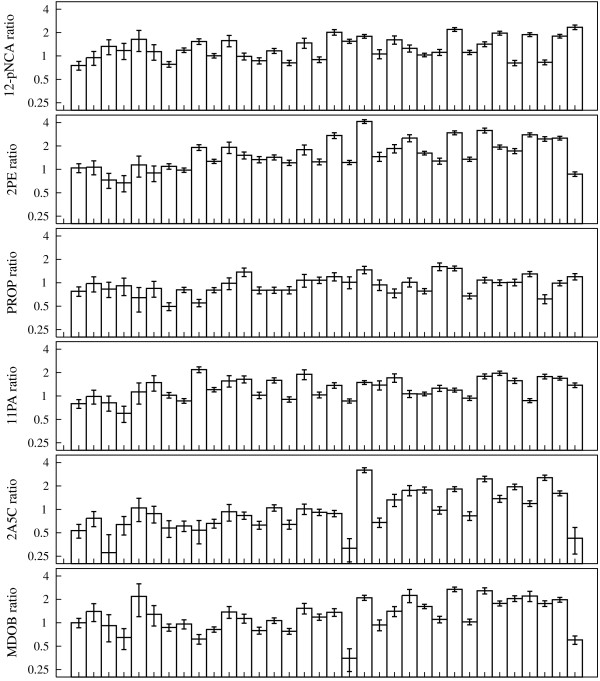
**Fold changes in P450 activities with standard errors**. The bar graphs show the fold change in activity of all 34 neutrally evolved P450 variants on all six substrates. This is the same data as in Figure 2, except these graphs also give error bars showing the standard errors in two separate measurements of the activities. In most cases the standard errors are much smaller than the activity changes themselves.

We measured the activities of all 34 neutrally evolved P450s on the five promiscuous substrates as well as 12-pNCA. Figure 2 shows the fold change in activity of each of the variants relative to the parent P450 on all six substrates, and Figure 3 shows the same data with standard errors (raw data are in Additional files 2 and 3). As is apparent from these figures, many of the neutrally evolved P450s have undergone changes in their activities that substantially exceeded the standard errors of the measurements (for a more detailed analysis of measurement variability see the Methods and Additional file 4). Even on 12-pNCA, some of the variants have undergone modest increases or very mild decreases in activity. The modest increases in 12-pNCA activity were unsurprising, since the parent P450 only hydroxylates 12-pNCA with about a quarter of the activity reported for a P450 engineered for maximal 12-pNCA activity [31]. Likewise, the mild decreases in 12-pNCA activity were due to the fact that during neutral evolution the P450s were only required to maintain this activity above a minimal threshold (75% of the total 12-pNCA conversion of the parent protein when expressed in *E. coli *[16]). The changes in the promiscuous activities were often much larger than those on 12-pNCA. For example, several of the neutrally evolved variants have undergone nearly four-fold increases in activity on one or more of 2PE, 2A5C, and MDOB. Other variants have experienced equally large decreases in one or more of the promiscuous activities. The changes in promiscuous activities are therefore quite substantial, although still much less than the 10- to 40-fold increases reported in directed evolution experiments that explicitly selected for improvement in a previously unselected P450 BM3 activities [31-33].

### Broad patterns of change in activity can be rationalized in terms of substrate properties

The data in Figures 2 and 3 clearly indicate that some of the P450s have undergone substantial changes in their activities. In an effort to understand the nature of these changes, we sought to determine whether there were any clear patterns in the activities. In Figure 2, the substrates have been hierarchically clustered so that each successive cluster contains substrates on which the P450s have increasingly similar activities (the clustering is illustrated by the tree-like dendrogram at the top of the figure, with similar substrates in adjacent columns). This clustering of the substrates based on P450 activities is also readily rationalized in terms of their chemical structures. For example, 2A5C and MDOB cluster, meaning that P450s with high activity on one of these substrates also tend to have high activity on the other. Presumably, they cluster because the similarity of their structures (both are fusions of six and five membered rings) means that they have similar modes of docking in the substrate binding pocket. Likewise, 12-pNCA and 11PA are phenoxycarboxylic acids of similar chain length, and are in the same cluster. To a lesser extent, 2PE resembles 12-pNCA and 11PA in its phenolic ether structure, and it falls into a higher level cluster with these two substrates. PROP shares a fused ring structure with 2A5C and MDOB, and these three substrates share a common higher level cluster. Overall, the hierarchical clustering indicates that substrates that appear similar to the human eye are also "seen" this way by the P450s, since the P450s tend to increase or decrease their activities on these substrates in a coordinated fashion.

Figure 2 also shows the P450 variants arranged in hierarchical clusters. A visual inspection immediately indicates that there is an overall association among all of the activities. Some of the P450 variants (redder rows) tend to show improved activity on most substrates, while others (bluer rows) tend to show decreased activity on most substrates. Taken together with the clustering of the similar substrates, this overall association suggests that there are two main trends in the activity changes. First, the P450s appear to have undergone general changes in their catalytic abilities that are manifested by broad increases or decreases in activity on all substrates. Second, the P450s appear to have experienced shifts in specificity to favor either the fused ring or the phenolic ether substrates.

To test whether these two apparent trends in activity changes are supported by a quantitative examination of the data, we performed principal component analysis. Principal component analysis is a well-established mathematical technique for finding the dominant components of variation in a data set, essentially by diagonalizing the covariance matrix. As suggested by the foregoing visual inspection, principal component analysis revealed that two components explained most of the changes in P450 activities (Table 1). The first component contained positive contributions from all six substrates, and so represents a general improvement in catalytic ability. The second component contained positive contributions from the fused ring substrates and negative contributions from the phenolic ether substrates, and so represents an increased preference for the former class of substrates over the latter. Together, these two components explain 82% of the variance in activities among the 34 P450 variants. The remaining 18% of the variance is explained by the four remaining components, which represent more subtle shifts in activity that are less easily rationalized by intuitive chemical arguments. Note that the major trends in activity changes (overall increase/decrease in catalytic ability, and increased preference for either phenolic ether or fused ring substrates) are unlikely to be caused by the occurrence of the same mutation in several P450 variants, since most of the variants share no common mutations (Additional file 1). Instead, there must be a number of different mutations that can contribute to each of these trends.

**Table 1:** Principal component analysis of activity profiles.

	12-pNCA	2PE	PROP	11PA	2A5C	MDOB
PC1 (explains 62% of variance)	0.25	0.61	0.05	0.29	0.47	0.51
PC2 (explains 20% of variance)	-0.48	-0.39	0.14	-0.27	0.70	0.19

The first two principal components explain 82% of the variance in P450 activity profiles. The table shows the composition of these two components and the variance explained by each. The first component contains positive contributions from all substrates and can be thought of as representing a general high catalytic ability. The second component can be thought of as representing discrimination between fused ring substrates (PROP, 2A5C, and MDOB) and phenolic ether substrates (12-pNCA, 11PA, 2PE). Substrate abbreviations are as defined in the legend to Figure 2. The analysis was performed on the logarithms of the fold changes in activity.

### Overall distributions of change in the activities

The preceding sections have demonstrated that neutral genetic drift can lead to substantial changes in P450 activities, and that many of these changes can be understood as resulting from either fairly general increases/decreases in catalytic ability or shifts in preference for different broad classes of substrate structures. In this section, we examine whether there are any pervasive trends in the distributions of activity changes – for example, did most of the promiscuous activities tend to increase or decrease? If a property is not under any evolutionary constraint, then during neutral genetic drift its values might be expected to be distributed in a roughly Gaussian fashion, as the neutrally evolving proteins freely sample from the presumably normal underlying distribution. On the other hand, if a property is constrained by selection to remain above a certain threshold, then during neutral genetic drift its values should display a truncated distribution since selection culls proteins with values that fall below the threshold (such a distribution has been predicted for protein stability by simulations [34] and theory [25]). In the case of our experiments, selection acted explicitly only on 12-pNCA activity, and changes in the other promiscuous activities were limited only by the extent that these activities were affected indirectly by this constraint (an effect termed "apparent selection" [35]).

**Figure 4:**
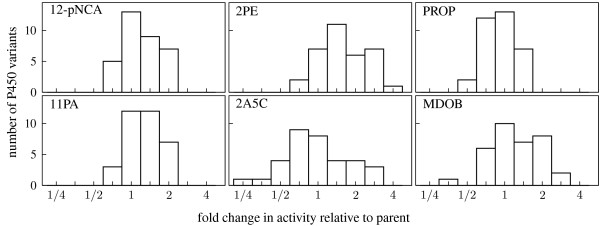
**Distributions of activity changes on each of the six substrates**. The histograms show the distributions of fold changes in activity for all 34 neutrally evolved P450 variants on each of the six substrates, with a value of one indicating that the activity is the same as the neutral evolution parent.

Figure 4 shows the distribution of changes in activity for each of the six substrates. The distribution for 12-pNCA appears to be truncated on the left, as expected since the P450s neutrally evolved under a requirement to maintain the ability to hydroxylate 12-pNCA. Some of the P450s have undergone a mild decrease in 12-pNCA activity, reflective of the fact that the neutral evolution selection criterion provided a small amount of latitude by allowing the total amount of hydroxylated 12-pNCA to drop to 75% of the parental value [16]. A number of P450s have neutrally evolved 12-pNCA activity that modestly exceeds that of the parent – again unsurprising, because the parental 12-pNCA activity falls well below the maximal value achievable for this type of protein [31]. The distribution for 11PA resembles that for 12-pNCA, probably because the activities on these two chemically similar substrates are highly linked, as discussed in the previous section. This is an example of apparent selection on one activity due to actual selection on a closely linked activity.

The other four promiscuous activities are less linked to 12-pNCA activity, and their distributions are much more symmetric. The symmetric shapes of these distributions suggest that neutral genetic drift has sampled from a roughly Gaussian distribution for these four promiscuous activities. For three of the substrates (PROP, 2A5C, and MDOB), the distributions of activities are approximately centered around the parental activity. This centering indicates that the promiscuous activities of the parent on these three substrates are typical of what would be expected of a neutrally evolved P450. The distribution for 2PE, on the other hand, is shifted towards activities higher than that of the parent. This shift indicates that the parent is less active on 2PE than a typical neutrally evolved P450, since most neutral mutations tend to increase this activity.

If the activity distributions of Figure 4 truly reflect what would be expected after a very long period of neutral genetic drift (i.e., if they are "equilibrium" distributions), then each variant represents a random sample from the underlying distribution of activities among all P450s that can neutrally evolve under this selection criterion. In this case, there should be no correlation between the extent of change in activity and the number of accumulated mutations, since the P450s should have lost all "memory" of the parent's activity. On the other hand, if there has only been a small amount of genetic drift, then the neutrally evolved P450s may maintain activity profiles that are similar to the parent simply because there have not been enough mutations to alter unselected properties of the enzyme (such as hydrophobicity properties of the active site that are not essential for the selected activity, but influence the promiscuous activities). In this sense, neutral genetic drift can be thought of as analogous to the equilibration of a physical system, since after a long period of time an equilibrated physical system will explore all microstates compatible with external constraints regardless of the initial condition, but after a short period of time the observed state may still resemble the initial condition.

In order to determine whether the neutral genetic drift had proceeded for enough mutations to "equilibrate" the activity profiles, we examined whether variants with fewer mutations more closely resembled the parent's activity profile. Such a resemblance would indicate that the neutral genetic drift had not yet completely eliminated residual memory of the parental activity. We computed the correlation between the magnitude of each variant's change in activity and the number of nonsynonymous mutations it possessed relative to the parent. Table 2 shows that the magnitude of activity change is positively correlated with the number of mutations for all six substrates. Although the correlations for the individual substrates are mostly not statistically significant due to the small number of samples, the overall correlation for all six substrates is highly significant (*P *= 10^-3^). Therefore, the P450 activities are still in the process of diverging from the parental values by neutral genetic drift. If the variants were to undergo further neutral genetic drift, one would expect to see even larger changes in their promiscuous activities. We note that the correlation between activity changes and number of mutations can be explained either by a model in which each successive mutation causes a progressively larger change in promiscuous activity, or by a model in which variants with more mutations are simply more likely to have experienced one of a relatively small number of mutations that lead to large changes in promiscuous activities.

**Table 2:** Correlations between changes in activity and number of mutations.

	12-pNCA	2PE	PROP	11PA	2A5C	MDOB	ALL
Correlation	0.32 (0.06)	0.27 (0.11)	0.06 (0.71)	0.12 (0.48)	0.20 (0.24)	0.36 (0.04)	0.22(10^-3^)

The extent of change in activity is positively correlated with the number of nonsynonymous mutations the P450 has undergone relative to the neutral evolution parent. Each column shows the Pearson correlation between the number of nonsynonymous mutations and the absolute value of the logarithm of the fold change in activity for a different substrate, computed over all 34 P450 variants. The final column (ALL) is the correlation among the 6 × 34 pooled data points for all six substrates. The *P*-values are shown in parentheses; none of the correlations for the individual substrates are significant at a 1% level (due to the small number of data points), but the overall correlation for all substrates is highly significant. Substrate abbreviations are as defined in the legend to Figure 2.

We also examined whether P450 variants with mutations near the substrate binding pocket were more likely to have undergone large changes in their activities. Five of the P450 variants had a mutation to a residue that was within 5 Å of the surrogate substrate in the P450 BM3 crystal structure [36]: variant M2 had A74V, M8 had A330V, M13 had M354I, M15 had A74P, and M24 had I263V [16]. Two of these mutated residues are of clear importance, since mutating residue 74 has previously been shown to shift substrate specificity [29,37,38] and residue 263 plays a role in the substrate-induced conformational shift [39]. We compared the activity changes for the five variants with mutations near the binding pocket to those for the 29 variants without any such mutations, computing the magnitude of activity change as the absolute value of the logarithm (base two) of the fold change in activity averaged over all six substrates. The average magnitude of activity change for the five variants with mutations near the active site was 0.88, while the average for the other 29 variants was 0.47. These averages are significantly different, with an unequal variance T-test *P*-value of 10^-2^. Therefore, variants with mutations near the substrate binding pocket have a higher probability of undergoing large changes in activity than all other variants, although many variants without mutations near the pocket also underwent substantial activity changes.

## Conclusion

We have shown that neutral genetic drift can lead to changes of as much as four-fold in the promiscuous activities of P450 proteins. The ubiquity of these changes is striking – even though many of the neutrally evolved P450s had only a handful of mutations, most of them had experienced at least some change in their promiscuous activities. P450s may be especially prone to this type of change, since their catalytic mechanism involves large substrate-induced conformational shifts [40] that can be modulated by mutations distant from the active site [32,37,41]. In addition, P450s have a tendency to eventually undergo irreversible inactivation that can be promoted by reduced coupling between substrate binding and conformational shifts, as well as by other poorly understood determinants of catalytic stability [19,42,43]. Therefore, the changes in P450 activities that we observed can be caused by factors that alter the catalytic lifetime of the enzyme in addition to factors that alter the maximum catalytic rate. There are thus ample opportunities for mutations that spread by neutral genetic drift to cause subtle alterations in a P450's promiscuous activities. But we believe that neutral genetic drift is also likely to cause substantial changes in the promiscuous activities of enzymes with other catalytic mechanisms. In support of this idea, a recent study by Tawfik and coworkers [44] indicates that mutations with little effect on the native lactonase activity of serum paraoxonase can alter this enzyme's promiscuous activities. Taken together, this study and our work suggest that promiscuous protein functions can change during neutral genetic drift. These changes could in turn have important implications for future functional evolution. For example, one can easily imagine a scenario in which neutral genetic drift enhances a promiscuous protein function, and then a subsequent gene duplication allows natural selection to transform one of the genes into the template for a protein with a full-fledged new functional role [1-6].

One of the most attractive aspects of our study is the degree to which the changes in P450 activities during neutral genetic drift could be understood in terms of the chemical structures of the substrates. Neutral genetic drift did not simply cause unpredictable shifts in activities. Instead, most of the variation was explained by two eminently intuitive components: an overall increase or decrease in catalytic ability, and a preference for either fused ring or phenolic ether substrates. We have suggested that neutral genetic drift under a fixed selection criterion can be viewed as sampling underlying "equilibrium" distributions of activities. The distributions for different activities are linked, since we have shown that P450s with good activity on one substrate will frequently also be highly active on chemically similar substrates (similar linkages have been observed in proteins created by recombination [45,46]). So while it may be impossible to know exactly how any specific mutation will affect a given activity, measuring a handful of activities may allow one to make relatively accurate predictions about other closely linked activities [47]. The prerequisite for making such predictions is an understanding of the linkages among activities in the set of sequences explored by neutral genetic drift. We have made the first steps in elucidating these linkages for P450s that have neutrally evolved under one specific selection regime. The linkages are very similar to those that would have been made by an organic chemist grouping the substrates on the basis of their chemical structures. Knowledge of these linkages is of use in understanding the origins of enzyme specificity [10,48] – if an enzyme displays high activity on one substrate but low activity on another, then either these two activities are negatively linked during neutral genetic drift or selection has explicitly disfavored one of them.

Our work also has implications for the general relationship between neutral genetic drift and adaptive evolution [49-54]. A number of studies focused on RNA [50-52] or computational systems [53,54] have suggested that genetic drift might aid in adaptive evolution. Our study and that of Tawfik and coworkers [44] support this notion for the evolution of new protein functions. However, the way that drift in promiscuous functions promotes adaptive evolution is slightly different than the paradigm proposed for RNA [50-52] and computational systems [53,54]. In those systems, neutral genetic drift is envisioned as allowing a sequence to move along its neutral network until it reaches a position where it can jump to a new higher-fitness and non-overlapping neutral network. In contrast, promiscuous protein functions change even as a protein drifts along a single neutral network. The adaptive benefits of this drift come when new selective pressures suddenly favor a previously irrelevant promiscuous function, in effect creating a new neutral network that overlaps with parts of the old one.

Overall, experiments have now demonstrated two clear mechanisms by which neutral genetic drift can aid in the evolution of protein functions. In the first mechanism, neutral genetic drift fixes a mutation that increases a protein's stability [24,25,55], thereby improving the protein's tolerance for subsequent mutations [26-28], some of which may confer new or improved functions [28]. In the second mechanism, which was the focus of this work and the recent study by Tawfik and coworkers [44], neutral genetic drift enhances a promiscuous protein function. This enhancement poises the protein to undergo adaptive evolution should a change in selection pressures make the promiscuous function beneficial at some point in the future.

## Methods

### Determination of P450 activities

We attempted to determine the activities of all 44 neutrally evolved P450 variants described in [16] (22 from the final monomorphic populations and 22 from the final polymorphic population). Ten of these variants expressed poorly in the procedure used here (as described in more detail below), and so were eliminated from further analysis since their low expression led to large errors in the activity measurements. That left activity data for the 34 neutrally evolved P450 variants listed in Figures 2 and 3, as well as for the R1-11 neutral evolution parent. The activities for each of these P450 variants were measured on all six substrates (12-pNCA, 2PE, PROP, 11PA, 2A5C, and MDOB). In all cases, the activities represent the total amount of product produced after two hours, and so are proportional to total substrate turnovers per enzyme. P450 BM3 enzymes typically catalyze only a finite number of reaction cycles before becoming irreversibly inactivated, and we believe that all reactions were essentially complete after two hours, so these activities should represent the total turnovers of the enzymes during their catalytic lifetimes.

To obtain P450 protein for the activity measurements, we expressed the protein using catalase-free *Escherichia coli *[56] containing the encoding gene on the isopropyl *β*-D-thiogalactoside (IPTG) inducible pCWori [56] plasmid (the catalase is removed since it breaks down the hydrogen peroxide used by the P450). The sequences of the P450 variants are detailed in [16]. We used freshly streaked cells to inoculate 2 ml cultures of Luria Broth (LB) supplemented with 100 *μ*g/ml of ampicillin, and grew these starter cultures overnight with shaking at 37°C. We then used 0.5 ml from these starter cultures to inoculate 1 L flasks containing 200 ml of terrific broth (TB) supplemented with 100 *μ*g/ml of ampicillin. The TB cultures were grown at 30°C and 210 rpm until they reached an optical density at 600 nm of ≈0.9, at which point IPTG and *δ*-aminolevulinic acid were added to a final concentration of 0.5 mM each. The cultures were grown for an additional 19 hours, then the cells were harvested by pelletting 50 ml aliquots at 5,500 g and 4°C for 10 min, and stored at -20°C. To obtain clarified lysate, each pellet was resuspended in 8 ml of 100 mM [4-(2-hydroxyethyl)-1-piperazinepropanesulfonic acid] (EPPS), pH 8.2 and lysed by sonication, while being kept on ice. The cell debris was pelleted by centrifugation at 8,000 g and 4°C for 10 minutes, and the clarified lysate was decanted and kept on ice.

To perform the assays, various dilutions of the clarified lysate were used to construct a standard curve. For each sample, we prepared dilutions of the clarified lysate in the 100 mM EPPS (pH 8.2) buffer to create samples for the standard curves. The dilutions were 100% clarified lysate (undiluted), 67% lysate, 40% lysate, 25% lysate, 17% lysate, 10% lysate, 6.7% lysate, and 4.0% lysate. Similar dilutions were also prepared of the clarified lysate of *E. coli *cells carrying a null pCWori plasmid in order to assess the background readings from lysate without any P450. A pipetting robot was then used to dispense 80 *μ*l of this series of clarified lysate dilutions into 96-well microtiter plates. Duplicate microtiter plates were then assayed for P450 concentration and total enzymatic activity on each of the six substrates. The R1-11 parent was assayed four times rather than in duplicate, with the replicates labeled A and B and those labeled C and D in Additional file 2 coming from two different protein expression cultures and lysis replicates. To minimize variation, all of these assays were performed in parallel, with the same stock solutions, and on the same day.

The P450 concentration was determined using the carbon monoxide (CO) difference spectrum assay [57]. Immediately before use, we prepared a 5× stock solution of 50 mM sodium hydrosulfite in 1.3 M potassium phosphate, pH 8.0. A multichannel pipette was used to add 20 *μ*l of this stock solution to each well of the microtiter plates (which contained 80 *μ*l of a dilution of clarified lysate), so that the final sodium hydrosulfite concentration was 10 mM in each well. The plates were briefly mixed and the absorbances were read at 450 and 490 nm. The plates were then incubated in a CO binding oven [57] for 10 minutes to bind CO to the iron. The absorbance was then again read at 450 and 490 nm. The amount of P450 is proportional to the increase in the magnitude of the absorbance at 450 nm minus the absorbance at 490 nm. At each dilution along the standard curve, the reading for the null control (lysate dilutions without P450) was subtracted from the reading for each P450 variant to control for clarified lysate background. Additional file 2 shows the standard curves for all P450 variants. Ten P450 variants had standard curve slopes less than or equal to 0.020, indicating a low P450 concentration. These were the ten P450 variants that we discarded from further analysis, since the low P450 concentration decreased the accuracy of the measurements.

To determine the activity on 12-pNCA, we monitored the formation of the yellow 4-nitrophenolate compound that is released upon hydroxylation of the twelfth carbon in the 12-pNCA molecule [31,58]. Immediately before use, we prepared a 6× stock solution of 12-pNCA by adding 3.6 parts of 4.17 mM 12-pNCA in DMSO to 6.4 parts 100 mM EPPS, pH 8.2. A multichannel pipette was used to add 20 *μ*l of this stock solution to each well of the microtiter plates (which contained 80 *μ*l of a dilution of clarified lysate). The plates were briefly mixed, and the absorbance was read at 398 nm. To initiate the reactions, we then prepared a 6× stock solution of 24 mM hydrogen peroxide in 100 mM EPPS, pH 8.2, and immediately added 20 *μ*l of this solution to each well of the microtiter plate and mixed. The final assay conditions were therefore 6% DMSO, 250 *μ*M 12-pNCA, and 4 mM hydrogen peroxide. The reactions were incubated on the benchtop for two hours, and the total amount of enzymatic product was quantified by the gain in absorbance at 398 nm. At each dilution along the standard curve, the corresponding null control lysate dilution was subtracted from the reading to control for lysate background. Additional file 2 shows the standard curves for all P450 variants.

The activities on 2PE, PROP, 11PA, 2A5C, and MDOB were determined using the 4-aminoantipyrene (4-AAP) assay [59,60], which detects the formation of phenolic compounds. For each of these five substrates, immediately before use we prepared a 6× substrate stock solution. These stock solutions were 6% DMSO and 6% acetone in 100 mM EPPS, pH 8.2, with an amount of substrate added so that the substrate concentrations in the stock solutions were: 150 mM for 2PE, 30 mM for propranolol, 5 mM for 11PA, 12 mM for 2A5C, and 120 mM for MDOB. The stock solutions were prepared by first dissolving the substrate in the DMSO and acetone, and then adding the EPPS buffer. In some cases, the stock solution became cloudy upon addition of the buffer, but there was no immediate precipitation, so we could still pipette the stock solution. A multichannel pipette was used to add 20 *μ*l of the appropriate substrate stock solution to each well of the microtiter plates (which contained 80 *μ*l of a dilution of clarified lysate). To initiate the reactions, we then added 20 *μ*l of the freshly prepared 6× hydrogen peroxide stock solution (24 mM hydrogen peroxide in 100 mM EPPS, pH 8.2) and mixed. We incubated the plates on the benchtop for two hours. To detect the formation of phenolic products, a pipetting robot was used to add and mix 120 *μ*l of quench buffer (4 M urea in 100 mM sodium hydroxide) to each well. We then used the robot to add and mix 36 *μ*l per well of 0.6% (w/v) of 4-aminoantipyrene in distilled water, and immediately read the absorbance at 500 nm. To catalyze formation of the red compound produced by coupling a phenolic compound to 4-aminoantipyrene [59,60], we then used the pipetting robot to add and mix 36 *μ*l per well of 0.6% (w/v) of potassium peroxodisulfate in distilled water. The plates were incubated on the benchtop for 30 minutes, and the amount of product was quantified by the gain in absorbance at 500 nm. At each dilution along the standard curve, the corresponding null control lysate dilution was subtracted from the reading to control for lysate background. Additional file 2 show the standard curves for all P450 variants.

In order to extract enzymatic activities from the standard curves, we fit lines to the data points. For some of the substrates (most notably 12-pNCA and 2PE), many of the P450 variants were sufficiently active to either saturate the substrate or exceed the linear range of absorbance readings. Therefore, we examined each standard curve by eye to determine which points remained in the linear range. Lines were then fit to the points in the linear range. These fits are shown in Additional file 2. In the plots in this file, all points that were deemed to fall in the linear range (and so were used for the fits) are shown as filled shapes, while all points that were deemed to fall outside the linear range (and so were not used in the fits) are shown as empty shapes. The figures show the slopes of the lines for all replicates (two replicates for all P450 variants except for R1-11, which had four replicates). These slopes are averaged for a best estimate of the slope, and the standard error computed over these two measurements is also reported.

To compare the activities (total substrate turnovers per enzyme) among the different P450 variants, it is first necessary to normalize to the enzyme concentration. To do this, we took the ratio of the slope for each substrate divided by the slope of the CO different spectrum, propagating the errors. These normalized slopes are proportional to the activity on each substrate. The normalized slopes are given in Additional file 3. This file also lists the number of nonsynonymous mutations that each P450 variant possesses relative to the R1-11 parent sequence, as originally reported in [16]. These normalized slopes allow for accurate comparisons among the P450 variants, and were used in the analyses in this paper. To convert these normalized slopes into total substrate turnovers per enzyme, it is necessary to multiply them by the ratio of extinction coefficients. The extinction coefficient for the CO difference spectrum reading (the absorbance at 450 nm minus that at 490 nm) is 91 mM^-1 ^cm^-1 ^[57], and we calculated the extinction coefficient at 398 nm for the 4-nitrophenolate group in our buffer to be 12,000 M^-1 ^cm^-1^. Therefore, for 12-pNCA, the total number of substrate turnovers per P450 enzyme is 7.58 times the ratio of the 12-pNCA standard curve slope to the CO difference spectrum slope. This indicates that our parent protein had about 250 12-pNCA turnovers per enzyme, compared to the 1,000 reported for a variant engineered for maximal 12-pNCA activity [31]. For the other substrates assayed with the 4-AAP assay, the extinction coefficient at 500 nm for the 4-AAP/phenol complex has been reported to be 4,800 [60]. However, we believe that this extinction coefficient could be of dubious accuracy for our data. Depending on the exact type of phenolic compound created by P450 hydroxylation, the extinction coefficient for the 4-AAP/phenol complex may vary. Assuming the extinction coefficient of 4,800 M^-1 ^cm^-1 ^is accurate, then the total number of substrate turnovers per P450 enzyme is 19.0 times the ratio of the substrate standard curve slope to the CO difference spectrum slope. Using this coefficient, the parent P450 had roughly 1,000 turnovers on 2PE, 30 turnovers on PROP, 400 turnovers on 11PA, 50 turnovers on 2A5C, and 80 turnovers on MDOB. The high activities on 2PE and 11PA are presumably due to the fact that lack of polar substituents on the aromatic ring allows these compounds to enter the hydrophobic P450 BM3 binding pocket [36] more easily than 12-pNCA. However, we emphasize that the exact numerical values for the turnovers for these five substrates are questionable. Definitive determination of the extinction coefficients would require analytical analysis of the enzymatic products for each P450 variant on each substrate, which is beyond the scope of this study.

### Analysis of activity data

The raw activity values computed for the P450 variants are listed in Additional file 3. To analyze and display this data, we computed the fold change in activity of each variant relative to the R1-11 parent P450. The fold change is simply the variant activity divided by the parent activity on each substrate, with the standard errors propagated to give an error on the fold change. In Figures 2 and 3, these fold changes are displayed on a logarithmic scale so that each unit corresponds to a two-fold increase or decrease in activity. In Figure 2, the substrates and the P450 variants have both been clustered, as shown by dendrograms on the side of the heat map. The clustering was performed using the standard hierarchical clustering function of the R statistical package. This is complete linkage hierarchical clustering, with the distances computed as the Euclidian distance between the logarithms of the fold changes in activity. The standard errors on the fold changes in activity are not incorporated into Figure 2 or any of the related analysis. However, these standard errors are shown in Figure 3; it is apparent from this figure that the errors tend to be much less than the fold changes in activity themselves.

In Figure 4, the histogram bins are logarithmically spaced so that each bin contains a 2^0.5^-fold range of activities. For example, the histogram bin centered at one contains all variants with between 2^-0.25 ^= 0.84 and 2^0.25 ^= 1.19 fold the parental activity, while the bin centered at 1.5 contains all variants with between 2^0.25 ^= 1.19 and 2^0.75 ^= 1.68 fold the parental activity.

The principal component analysis shown in Table 1 was performed using the R statistical package, with inputs being the logarithms of the fold changes in activity. Since these log fold changes in activity contained no arbitrary units (they were already normalized to the parent), the data was neither scaled nor zeroed before performing the analysis. Table 1 shows the composition and the percent of variance explained (the eigenvalue for that component divided by the sum of all eigenvalues) for the first two components. The remaining four components were relatively unimportant, explaining 7%, 5%, 4%, and 2% of the total variance.

### Variability in experimental measurements

Figures 2 and 3 show the changes in activity for each of the 34 neutrally evolved P450s. The data in this figure are the mean of two separate measurements performed on protein samples from the same preparation of expressed and lysed protein. Each of the two measurements involves independent determination of the P450 concentration and the enzymatic activity via the standard curves shown in Additional file 2. The two measurements are not fully independent biological replicates, since they come from the same preparation of protein. However, expression and lysis typically introduces variability in measurements due to differing concentrations of protein, and since each of the two measurements independently determines P450 concentration, they should correct for most of the variation induced by expression and lysis. To confirm this fact, and to obtain a more detailed characterization of the variability in the readings, we performed measurements on different preparations of the R1-11 parent protein. Overall, we used protein from four separate expression/lysis preparations of this protein. For each of these preparations, we performed two separate measurements of P450 concentration and activity. The resulting data are shown in the plots of Additional file 4, which is scaled so that it can be directly compared to Figure 3. The plots of Additional file 4 demonstrate that the variability in measurements on samples from different expression/lysis preparations is no larger than the variability between measurements from the same preparation. This confirms that the independent determination of P450 concentration for each measurement adequately controls for any differences between expression/lysis preparations. Additional file 4 also demonstrates that the overall variability in repeated measurements on the parent sample is small compared to the difference between samples, and is adequately captured by the error bars shown in Figure 3. So overall, while repeated measurements on the same sample do show a small amount of variability, this variability is smaller than the typical differences between different P450 variants, and is adequately captured by the duplicate measurements from the same preparation of protein that were used in this study.

### Phylogenetic tree

The phylogenetic tree shown in Figure 1 is based on the number of nonsynonymous mutations the P450 variants have relative to the R-11 neutral evolution parent. Each of the P450s that evolved in a monomorphic population (prefix of M) are known to have diverged independently, and so are drawn on their own branch regardless of any sequence identity to other variants. The exact phylogenetic relationship of the P450s that evolved in the polymorphic population (prefix of P) is not known, so the portion of the tree for these mutants was reconstructed by maximum parsimony. The tree is based only on the nonsynonymous mutations, and all mutations weighted equally. Full nucleotide and amino acid sequences of the P450s are given in Additional file 1.

## Authors' contributions

JDB, PR, and FHA designed the study. JDB, PR, and ZL performed the experiments. JDB and PR analyzed the data. JDB and FHA wrote the paper.

## Reviewers' comments

### Reviewer 1: Dr. Martijin Huynen, Nijmegen Center for Molecular Life Sciences and Center for Molecular and Biomolecular Informatics

The manuscript by Bloom et al presents experimental data to show how neutral evolution can explore phenotype space and help in adaptation, adding a new answer to Sewall Wright's question to Kimura about the potential "future" benefit of neutral evolution [61]. They show that the proteins that are more or less equivalent with respect to their primary function, and that are connected through a neutral path, vary with respect to their ability to perform "other", promiscuous functions for which there has been no selection. The manuscript is well written, the results speak for themselves, the arguments are sound, and I do not have much to add. I should comment that I am not an experimentalist, and can therefore not judge the experimental results in detail.

#### Authors' response

*We thank the reviewer for the paragraph putting the manuscript in this context of Wright's question to Kimura*.

Specific questions:

Can the "four-fold" change in the promiscuous activities be put into perspective? How does this e.g. compare to the activity of a protein that has specifically been selected for any of the tested functions?

#### Authors' response

*This is an excellent question. The short answer (which is now briefly mentioned in the main text) is that studies selecting for improvement P450 BM3 activities that have not previously been the target of selection tend to report increases of 10- to 40-fold. As a caveat, we note that none of these studies exactly parallel the experimental methodology used here. One study that selected for high P450 peroxygenase activity on the substrate 12-pNCA increased the total substrate turnovers 11-fold with 9 amino acid mutations (not all 9 mutations necessarily contributed to the improved activity; some may have "hitchhiked" along) *[31]. *A study beginning with the P450 BM3 holoenzyme increased the maximal rate of octane hydroxylation 38-fold with 9 amino acid mutations *[32]. *Another study beginning with a P450 holoenzyme variant increased the number of turnovers of propane hydroxylation 12-fold with a dozen amino acid mutations *[33].

Can the variation in catalytic activity also be explained as a variation in enzyme catalytic lifetimes?

#### Authors' response

*Yes! By measuring total substrate turnovers, we are essentially measuring the product of the catalytic rate times the catalytic lifetime. P450s are known to have finite catalytic lifetimes that depend on the degree of coupling between substrate binding and the conformational shifts associated with the enzyme catalytic cycle. So mutations could affect the catalytic rate, the overall catalytic lifetime of the protein, or the catalytic lifetime of the protein on a particular substrate due to the degree of coupling between binding to that substrate and conformational changes. The fact that the total substrate turnovers depend on this entire set of protein properties is probably the reason why so many different mutations influence activity*.

Is it possible to separate the two effects of the mutations on the activity (substrate specificity versus "overall" catalytic activity) by where those mutations occurred in the protein, like you do in the last paragraph of the Results section by averaging the effects of the mutations for all 6 substrates?

#### Authors' response

*Unfortunately, we do not believe that it is possible to convincingly make such a separation. The reason is that P450 is a very dynamic enzyme that undergoes large conformational changes during its catalytic cycles – indeed, the exact structure of the catalytically active form is not even known (the substrate position in the crystal structure conformation is thought to place the substrate several angstroms too far from the iron for catalysis). Therefore, mutations that are distant from the active site have on occasion been shown to affect substrate specificity. So there is no clear basis for predicting which mutations contribute more to overall activity versus specificity*.

Fourth paragraph of the "Overall distributions of change in the activities" in Results: "On the other hand if there has not been enough neutral genetic drift etc." Could you change that sentence in one way or another such that it becomes clearer what is what relative to what? e.g. "On the other hand if there has not been enough neutral genetic drift after a few mutations to completely eliminate etc."

#### Authors' response

*We have clarified this point. There are some properties of the protein that are not essential for the selected activity but that influence the promiscuous activities. Neutral genetic drift will tend to change these properties, and after some number of mutations the properties will have changed enough that they are no longer correlated with the properties of the evolutionary parent more than would be expected for any two random proteins that satisfied the selection criterion. Our original meaning was that there had been "enough" mutations that this correlation was gone. In analogy with physics, this would occur when the autocorrelation function had decayed to zero. In any case, we have rewritten the relevant sentences to make clear that "enough" means that the correlation between the parental and neutrally evolved proteins is no larger than between a random pair of proteins that satisfy the selection criterion*.

### Reviewer 2: Dr. Fyodor Kondrashov, University of California San Diego

Investigations and descriptions of fitness ridges (a fitness ridge is a function relating fitness and genotype) are necessarily difficult and confusing due to the complex and often multidimensional nature of such ridges. Thus, it is truly remarkable that in the recent past several studies have attempted to describe the shapes of fitness ridges one gene at a time. The present study is one of them. The authors study the effects of mutations on several promiscuous functions that have been selected for not ruining the activity of a related but distinct function. Indeed, under laboratory conditions the mutations that are allowed to propagate are neutral by definition, and the authors in essence studied the effect of mutations accumulated by drift. However, I do not think that the main claim of this paper, that "drift can aid functional protein evolution" is appropriate and I believe that the results are much more important to the study of fitness ridges than to the study of the impact of neutral drift.

Assuming a starting condition of such neutral evolution when the activity of promiscuous functions truly bears no fitness effects, consider the impact of the past several mutations right before this activity became a target of selection. In an equilibrium setting, those mutations are equally likely to have decreased the level of the activity as they are to have lead to an increase. Therefore, the paper could have been titled "drift can inhibit functional protein evolution" just as well. Regardless, I think that the impact of such an artificially neutral mutation on future adaptations is not the issue here. Just as with the original work by Aharoni et al. [2], the issue is the degree of pleiotropic interactions of different functions that can be performed by a single protein because understanding the nature of such interactions is crucial for our understanding of fitness ridges.

#### Authors' response

*The reviewer raises two main points. The first point is that neutral genetic drift can both increase and decrease promiscuous functions. The reviewer therefore argues that the original title of our manuscript, "Neutral genetic drift can aid functional protein evolution," was misleading since this drift can also decrease promiscuous functions. We did not intend for this title to convey the idea that genetic drift always increases promiscuous functions. Rather, we meant that such drift will in some cases increase promiscuous functions, and that these increases can aid functional evolution if there is subsequently selective pressure that favors an enhanced promiscuous function. Essentially, genetic drift is increasing the overall variance in promiscuous functions – something that will be beneficial for functional evolution if subsequent selection amplifies those variants with enhanced function. To better convey this idea we have now changed the title of the paper to "Neutral genetic drift can alter promiscuous protein functions, potentially aiding functional evolution." The reviewer's second major point is that our work can be interpreted in terms of fitness landscapes rather than in terms of neutral genetic drift. We have added a text mentioning how our work sheds light on some of the underlying relationships among different protein functions in the fitness landscape. The reviewer also suggests that we interpret our results in terms of "fitness ridges" rather than "neutral drift" on "neutral networks." Essentially, a fitness ridge *[23]*is a connected set of high fitness genotypes. When all genotypes are classified as either functional or nonfunctional (as in our experiments), a fitness ridge is the same as a neutral network because selection does not distinguish between points on the ridge of different heights. We now explain this in the text. However, in our experiments we do not know the true fitness function for P450 BM3 enzymes evolving in nature (this would require full knowledge of the complex interactions among the protein, the bacteria's metabolism, and a potentially fluctuating environment), and so we cannot confidently map our measurements of P450 enzymatic activities to the fitness landscape. Therefore, in our evolution experiments we defined a simplified fitness function in which P450s are "functional" if their activity on 12-pNCA exceeds a threshold, and "nonfunctional" otherwise. This is clearly not the real natural fitness function, but it provided an abstraction of the basic idea that enzymatic activity must meet some minimal threshold for bacterial viability. In the context of our experiments, we therefore feel that neutral networks and neutral drift provide an appropriate description of the types of mutations that have occurred. The reviewer is nonetheless correct that it might also be possible to use our results to map features of the P450 fitness landscape; although we do not attempt this interpretation ourselves, we have provided the full raw data from our experiments in case others are interested in doing so*.

The work Aharoni et al. [2] suggested that if a new previously unused function is suddenly placed under the spotlight of selection, such new functions can be found (and substantially improved) in proteins that are already are doing something else. The present work strongly supports this assertion. The authors also found that the effect of mutations on the original function is correlated to the level of similarity of the promiscuous functions. This observation adds a very interesting and counter intuitive caveat: if the activity of the native function cannot be altered, selection has a better chance improving those functions of a gene that are more different from the original function. Obviously life is not this simple and there must be a limit to how much a promiscuous function can differ from the original. However, evolutionary examples of enzyme recruitment in the vertebrate eye lens or the evolution of antifreeze proteins have already given the proof of concept that novel functions can evolve from seemingly inappropriate genes.

#### Authors' response

*We thank the reviewer for the observations contained in this paragraph, and have added some of them to the text of the manuscript*.

Secondary comments: Second paragraph of Background section. I disagree that it is clear that only a small fraction of mutations in proteins are adaptive. For an example see: Andolfatto P. Adaptive evolution of non-coding DNA in Drosophila. Nature. 2005 Oct 20;437(7062):1149-52.

#### Authors' response

*We have rephrased the offending passage. We did not intend to suggest that most natural mutations are necessarily due to neutral genetic drift, since we recognize that this is a hotly debated topic. Instead, we simply meant that most mutations are not driven by selective pressure for an entirely new function, since the basic biochemical functions of most proteins are relatively stable over long periods of evolutionary time even as many mutations accumulate (as pointed out by Zuckerkandl and Pauling *[13]*and many subsequent studies). It is of course hard to discern whether these mutations are due to neutral genetic drift or some more subtle selection pressure not due to a wholesale change in protein function (as suggested by *[15]). *In any case, we have toned down the relevant passage to merely convey the idea that proteins accumulate many mutations that are either due to genetic drift or very subtle selection pressures unrelated to evolution of an entirely new function*.

I think that one of the first papers on the impact of neutral mutations for future adaptations has been: Lipman DJ, Wilbur WJ. Modelling neutral and selective evolution of protein folding. Proc Biol Sci. 1991 Jul 22;245(1312):7–11.

#### Authors' response

*We thank the reviewer for bringing this reference, which is based on computational lattice models of proteins, to our attention. It is now cited in our work*.

First paragraph of "Overall distributions of change in the activities" section of Results: Another factor that may greatly affect whether or not mutations that change a promiscuous function are at equilibrium is whether or not there is selection against such function. Perhaps such selection is expected, because a promiscuous function may act as a competitive inhibitor of the original one. The possibility of such selection is another reason why I think that although in the laboratory these mutations are by definition neutral, we must first study the fitness function of these proteins before investigating the importance of drift.

#### Authors' response

*We agree with the reviewer that in a natural setting there may be selection against some promiscuous functions, and we now mention this possibility in the text. However, due to the limitations of what is realistically possible to accomplish in the laboratory, we are not in a position to determine the natural fitness function for P450 enzymes. The reason is that while it is possible to accurately measure enzymatic activities as we have done in this paper, we do not have the capability of directly measuring how these changes in activity would affect the fitness of an organism in a natural setting*.

First and second paragraphs of "Overall distributions of change in the activities" section of Results: When selection on one phenotype causes a change in another, this is called "apparent selection."

#### Authors' response

*We thank the reviewer for pointing out the correct term for this phenomenon; we now use this term at the relevant point in the text*.

Third paragraph of Conclusions: "The adaptive benefits of this drift come when new selective pressures suddenly favor a previously irrelevant promiscuous function, in effect creating a new neutral network that overlaps with parts of the old one." If a new selective pressure appeared, it changed the neutral network to a ridge. In general I think that the term "neutral network" should be avoided because it describes a specific model where each mutation is either lethal or neutral. This is exactly replicated in this experimental setup, however, as soon as adaptive selection comes into play the "neutral network" model becomes insufficient to describe either the fitness states of each genotype or the evolutionary processes that follow. In general, the term "fitness ridge" or "fitness landscape" is preferable for this reason.

#### Authors' response

*This point is addressed to some degree in previous responses to the reviewer's comments. Essentially, we have now comment on the relationship between fitness ridges *[3]*and neutral networks. However, we have still retained the term "neutral network" in the text, since this term is widely used and since we do not know the true fitness function of P450s (the binary selection of our experiments map fitness ridges to neutral networks)*.

I would be interested to see the correlations of the change of each promiscuous function to that of the native one. Figure 2 and 3 but with the values showing a correlation. Just by looking at Fig 2, it seems that every single function is actually correlated, but it would be interesting to see how much.

#### Authors' response

*The reviewer is correct that the promiscuous activities are correlated to the native one. However, rather than computing correlation coefficients we have chosen to perform the principal component analysis shown in *Table 2. *The reason is that this sort of analysis better captures the complete relationship among all six functions, which contains extensive covariances. One major component of this relationship is indeed the correlation among all activities. However, another major component of the relationship is the tendency of the P450 to favor either the phenolic ether or fused ring substrates. These two relationships tend to obscure each other when analyzed in terms of simple correlation coefficients, so for this reason we prefer the principal component analysis*.

### Reviewer 3: Dr. Dan S. Tawfik (nominated by Christoph Adami), Weizmann Institute of Science

Major comments:

1. Outside the context of organismal fitness, neutrality is obviously hard to define, and even the former is highly dependent on growth conditions. The limitations of determining neutrality in an in vitro system should be discussed. These limitations should also be addressed in view of the fact that P450s are broad range, rather than a 'one substrate' enzyme. Evidence in favour of 12-pCNA being a reasonable surrogate of the primary substrate(s) of the P450 studied here should also be outlined.

#### Authors' response

*This is an excellent comment by the reviewer (some similar points were also raised by Reviewer 2) that is now addressed in more detail in the main text. Quite simply, a limitation of our study is that we lack a solid basis for connecting the changes we observe in P450 activity to organismal fitness. We do not know exactly how a modest change in the activity profile of its P450 enzymes would affect the metabolism of a bacterium living in an environment with fluctuating concentrations of various substrates. As the reviewer points out, P450s tend to be broad range, and catalyze multiple reactions in biological settings. In fact, the primary natural substrates of P450 BM3 are not definitively known, although they are thought to include fatty acids *[19,20]. *There is thus no strong basis for believing that 12-pNCA is a close surrogate of a primary natural P450 BM3 substrate, and the reviewer is completely correct that natural P450s are probably not one-substrate enzymes. We therefore make no claims that the evolutionary changes that we observe in our studies parallel any specific changes that would be of biological relevance in natural P450 BM3 evolution. Instead, our study relates to the more general issue of how different enzyme specificities can evolve – certainly, such evolution is of relevance in biology, even if we cannot tie our exact choice of substrates to those that occur in nature. Our choice to define mutations as "neutral" if they maintain 12-pNCA activity above some threshold is based on the abstraction that selection in nature requires specific P450 activities to exceed some minimal value for viable metabolism. Given this abstraction, we believe our study sheds valuable light on how promiscuous activities can change when enzymes are constrained by limits on another primary activity*.

2. Being an integral part of the Results section, the sequence of the studied variants should be provided for this paper (notwithstanding [16], e.g., in the form of a table listing all mutations.

#### Authors' response

*We have added a text file as *Additional file 1*that lists the mutations of all the neutrally evolved P450 variants as well as the full sequence of the parent P450*.

3. Given the nature of the drift that led to the studied variants, the phrase "any mutations that spread among offspring sequences were therefore by definition due to neutral drift..." (second paragraph of Results section), should be supported by showing that the mutations in the studies variants all largely polymorphic, and none was enriched/fixated (see also point 2 above).

#### Authors' response

*As mentioned above, we have added a list of all mutations *(Additional file 1). *In a few cases, the same mutation occurs in more than one P450 variant. However, we see no strong evidence of any specific mutation being unusually enriched among any of the variants. Overall, of the 105 different amino acid mutations occurring in the set of P450 variants, only twelve occur in more than one variant. Of these twelve mutations that occur multiple times, six are found mostly in variants from the polymorphic population that are evolutionarily related on the phylogenetic tree of *Figure 1*(for example, P9, P13, and P18 share a common mutation, but are evolutionarily related), meaning that the multiple occurrences can be explained by neutral spread of a single mutation to several members of the polymorphic population, rather than by repeated occurrence and fixation of the same mutation. So a visual inspection of the mutation data does not indicate any glaring trends for the enrichment of specific mutations. We have not attempted a formal population genetics analysis to see if the frequency distribution of mutations is compatible with the neutral theory (such an analysis would be complicated by the need to correct for phylogenetic relationships and the fact that error-prone PCR is biased towards creating certain types of mutations). However, our inclusion of the full mutational data should allow the reader to attempt such a population genetic analysis if he/she desires to do so. In any case, our statement that any mutations are "by definition due to neutral genetic drift" is simply based on the fact that the experimental procedure cannot directly favor any mutation, since all mutants have either equally high or zero fitness*.

4. The standard errors refer to two separate measurements of the activities (legend of Figure 3). However, the Methods section describes a protocol by which a single lysate was aliquoted to two plates and measured separately (third paragraph of Methods section). This procedure hardly reflects the major sources of experimental variability, namely, differences in growth densities and lysis efficiency. Thus, separate measurements should involve independent growth of two colonies, lysis, and activity measurements.

#### Authors' response

*This comment is addressed along with comment 5 in the next response*.

5. There is no proper assessment of the likelihood that the observed variations in the measured activity are simply due to experimental variations. In other words, data should be provided to indicate the level of variation observed in an appropriate set of independent repeats of the parental protein ("wild-type") grown, lysed, and assayed for the various activities.

#### Authors' response

*This response addresses the last two comments from the reviewer (4 and 5). New data and discussion to address these comments has been added in the form of *Additional file 4*and the new Methods section "Variability in experimental measurements." Here we briefly recap this new data and discussion. The reviewer correctly notes that the duplicate measurements of activity were performed on P450 from the same expression/lysis preparation of protein, and so do not constitute full biological replicates (which would start with independent single bacterial colonies). However, most of the variation due to expression/lysis is due to differences in enzyme concentration in the resulting lysate. In most high-throughput assays, this is a severe problem since there is no means for correcting for differences in enzyme concentration. But in the case of our experiments, for each measurement we constructed a full standard curve to accurately determine the P450 concentration as well as the activity *(Additional file 2), *thereby correcting for any variability in P450 concentration. To demonstrate that these measurements capture the variability between different measurements, we now describe activity measurements on four separate expression/lysis preparations of the parental protein (as suggested by comment 5 from the reviewer). These measurements are now shown in *Additional file 4. *They show that the variation between different expression/lysis preparations is no larger than the difference of separate measurements on samples from the same preparation, confirming that the approach we have taken adequately corrects for most of the experimental variability. The data in *Additional file 4*also show that the experimental variability of repeated measurements on different preparations of parental protein is small compared to the typical differences among different P450 variants. Overall, these data support the notion that the error bars in *Figure 3, *calculated from separate measurements from the same expression/lysis preparation and containing propagated errors from repeated measurements on the parental protein, are a fair representation of the experimental variability*.

Other comments:

1. One of the questions remaining to be answered concerns the relationship between the level of neutrality that has to be maintained and the changes and improvements displayed in the promiscuous activities. This study shows that the original activity can be maintained while exhibiting changes in promiscuous activity. However, the issue regarding a possible tradeoff between the two still remains unknown. It is therefore worthwhile, using the results from this study, to check if there is any form of association between the level of 12-pNCA activity, for each variant, and the tendency to show large deviation in other promiscuous substrates.

#### Authors' response

*The reviewer raises the excellent question of whether the P450s are able to change their promiscuous activities by large amounts while still maintaining 12-pNCA activity. To look at this question, we examined the correlation between fold-change in 12-pNCA activity and the extent of change (absolute value of the logarithm of the fold-change in activity) on each of the five promiscuous substrates. The correlations and their P-values are: 0.51 (0.003) for 2PE, -0.07 (0.67) for PROP, 0.29 (0.10) for 11PA, 0.07 (0.66) for 2A5C, and 0.53 (0.002) for MDOB. Therefore, only two of the promiscuous substrates (2PE and MDOB) show a significant positive correlation between 12-pNCA activity and the amount of change in promiscuous activity. However, these correlations do not necessarily indicate an underlying relationship between 12-pNCA activity and amount of change in promiscuous activity. The reason is that, as shown in *Table 2, *the extent of change in all promiscuous activities tends to increase with more mutations, and this effect is largest for 2PE and MDOB. The change in 12-pNCA activity also increases with the number of mutations *(Table 2). *Therefore, it seems likely to us that the correlation between 12-pNCA activity and the change in promiscuous 2PE and MDOB activities is simply due to the fact that changes in all of these activities increase with more mutations. In order to control for this effect, it would be necessary to look at activity changes in a set of proteins with roughly the same numbers of mutations. Unfortunately, the iterative nature of the evolution experiments that we used to create our P450 variants created a large variance in the number of mutations per variant. Therefore, our study is not well posed to answer this question more quantitatively. However, we do refer the reader to *[44]*for a study that is better designed to answer this question*.

2. The relationship between the number of mutations and the degree of improvement toward promiscuous activities can be explained also by a probability distribution that describes the number of successes in a sequence of n draws from some finite population (e.g. hypergeometric distribution). For example, assume there is a small set of residues in P450 that influences protein activity upon mutation. In such a case, the higher the number of mutations imposed on the protein, the more likely the event that we randomly pick up one or more of the residues from that small set. In such a scenario, a large number of mutations does not form the basis and is not a prerequisite for a change in activity. Rather, it is just related to the elevating the probability of picking the influential residues by random mutagenesis.

#### Authors' response

*The reviewer is absolutely correct that the correlation between the number of mutations and the extent of activity is probably due to the fact that the occurrence of more mutations increases the chance that one of these mutations is at an influential residue. We believe our interpretation of the data is consistent with this explanation, and have slightly modified the text to emphasize this point along the lines suggested by the reviewer. However, we do not speculate on the exact distribution of activity changes caused by random mutations, since there are probably some highly influential mutations, some moderately influential ones, and some with essentially no effect*.

3. Do the specific clusters of variants shown in figure 2 relate to specific mutation, or a group of specific mutations? Since the number of variants analyzed is relatively small, it would be best to add a table showing the genotype of each mutant.

#### Authors' response

*The clustering of variants in *Figure 2*is based on their activity profiles (variants with similar activity profiles are clustered together). Overall, the sharing of common mutations does not appear to be a major determinant of these activity profile clusters. The reason is that most variants do not have any mutations in common (of the 105 different amino acid mutations, only 12 occur in more than one variant)*. Figure 1*captures some of the mutational clustering of the variants. In a few cases, variants that are evolutionarily related *(Figure 1) *also cluster together based on activity *(Figure 2), *for example P16 and P22. But there are also cases of variants that share an evolutionary relationship that do not cluster in activity, for example P8 and P10. We have not done a formal analysis of the relationship between common mutations and activity clustering for the simple reason that most variants share no common mutations with any other variant, and so cannot be meaningfully included in such an analysis. We interpret the fact that there are similarities in activity profiles despite little sharing of common mutations to indicate that many different mutations can cause similar changes in activity profiles (for example, there are multiple different mutations that preferentially enhance activity on either phenolic ether or fused ring substrates). In any case, as suggested by the reviewer, we have now included a table *(Additional file 1) *listing the full genotype of each variant. We also now briefly make reference to this issue in the text*.

4. The distribution of 2-phenoxyethanol in Figure 4 is skewed to the right. The authors mention that it indicates that the parent is less active on that particular substrate than a typical neutrally evolved P450. On what grounds is that claim given? Is there any supporting data on other neutrally evolved P450 to support that? If not, could it be that the sequence context of R1-11 promotes improvements towards 2-phenoxyethanol because of some specific position on the neutral network that elevate the chances of increasing activity against 2-phenoxyethanol?

#### Authors' response

*The only grounds for claiming that the parent is less active on 2-phenoxyethanol than the typical neutrally evolved P450 is the fact that most of the neutrally evolved P450s had higher 2-phenoxyethanol activity than the parent. There are no supporting data from other studies (since we know of no similar studies on P450s). In any case, we believe that saying that the parent is less active on 2-phenoxyethanol than the typical neutrally evolved P450 is essentially the same as saying that the parent occurs on a position of the neutral network that elevates the changes of increasing activity on 2-phenoxyethanol. That is, if the parent is at a neutral network position with abnormally low 2-phenoxethanol activity, most nearby sequences on the neutral network would be expected to have higher 2-phenoxyethanol activity*.

Comments regarding style:

1. The term "ask new questions" (last sentence of abstract) is somewhat colloquial. Wouldn't "favour new functions or metabolic capabilities" be equally clear?

#### Authors' response

*We have accepted this recommendation, and replaced "ask new questions" with "favor new functions*."

2. Why aren't substrate names abbreviated all along the article?

#### Authors' response

*We were hoping to minimize the use of confusing abbreviations, but have realized that our mixing of abbreviations and full names was probably even more confusing. Therefore, we have accepted the recommendation to abbreviate the substrate names throughout the article after defining the abbreviations at their first appearances in the text and in the figures*.

3. In the sentence: "Therefore, variants with mutations near the substrate binding pocket are especially likely to have altered activities" author may change to: "Therefore, variants with mutations near the substrate binding pocket show higher probability of attaining large differences in activity as compared to all other variants."

#### Authors' response

*We have accepted this recommendation, since it more precisely conveys the meaning we intended*.

4. In the Methods section under the header "phylogenetic tree" fix the sentence (the word "they" specifically): "...polymorphic population (prefix of P) is not known so they portion of the tree for these mutants..."

#### Authors' response

*We thank the reviewer for pointing out this error, which we have corrected*.

5. In the legend for Table 1 correct "...phenolic either substrates..." to "phenolic ether substrates."

#### Authors' response

*We thank the reviewer for pointing out this error, which we have corrected*.

6. In figure 1, variant P9 is shown to be at the top of a vertical branching line. What does it mean? It is probably some mistake.

#### Authors' response

*It is actually not a mistake. The horizontal lengths of the branches indicate the number of accumulated mutations, while the vertical spacing is arbitrary, and simply chosen to yield a tree that does not look too crowded. In the specific case of variant P9, P9 shares a common mutation with P18 and P13 but has only this one mutation while P18 and P13 also have other mutations. Therefore, P9 should be placed at the point in the tree at which P18 and P13 diverge. In order to indicate that it is at this location, the tree-drawing program draws a vertical branch for P9. The legend explains that the vertical spacing is arbitrary while the horizontal length is proportional to the number of accumulated mutations. We see how this could be somewhat confusing, but it is a standard feature of trees drawn in this fashion, and we are not sure of a better way to indicate the position of P9*.

## Supplementary Material

Additional file 1 **Mutations in neutrally evolved P450s**. The text file gives the full sequence of the P450 R1-11 neutral evolution parent, as well as the individual mutations of all 34 neutrally evolved P450 variants. These data were originally reported in [16]. Click here for file

Additional file 2 **Standard curves used to determine P450 activities**. The PDF file shows all of the standard curves used to determine the P450 concentration and enzymatic activities. Points that were deemed to fall in the linear range, and so used to compute the standard curve slopes, are solid. Points that were deemed outside of the linear range are empty. Each curve shows the slopes computed for two different measurements of P450 concentration and enzymatic activity, and the average slope with standard error. These average slopes were used to compute the P450 activities. Click here for file

Additional file 3 **Raw activity and sequence data**. The text file gives the activities of the P450 variants on each of the six substrates, as measured in this study. It also lists the number of nonsynonymous mutations (M_NS) relative to the R1-11 neutral evolution parent sequence, as originally reported in [16]. Each row lists the data for a different P450 variant, and standard errors for the measured activities are shown in parentheses. Activities are the normalized standard curve slopes as described in the Methods. Click here for file

Additional file 4 **Variability of experimental measurements**. The attached PDF file shows the results of repeated activity measurements performed on the R1-11 parent P450. The plots are scaled so that they can be directly compared to Figure 3 to see how much of observed differences among neutrally evolved P450 variants might be due to variability in experimental measurements. Such a comparison indicates that the experimental measurement variability is much less than the differences between P450s. The plots also show that most of the experimental variation is introduced during the activity measurements rather than during expression/lysis. Click here for file
