# Supplementary material for: Neutral genetic drift can alter promiscuous protein functions, potentially aiding functional evolution
Source: Biol Direct. 2007 Jun 28;2:17. doi: 10.1186/1745-6150-2-17 (PMC1914045; doi:10.1186/1745-6150-2-17)
Supplement: Additional file 2 — Standard curves used to determine P450 activities. The PDF file shows all of the standard curves used to determine the P450 concentration and enzymatic activities. Points that were deemed to fall in the linear range, and so used to compute the standard curve slopes, are solid. Points that were deemed outside of the linear range are empty. Each curve shows the slopes computed for two different measurements of P450 concentration and enzymatic activity, and the average slope with standard error. These average slopes were used to compute the P450 activities. [file 1745-6150-2-17-S2.pdf]

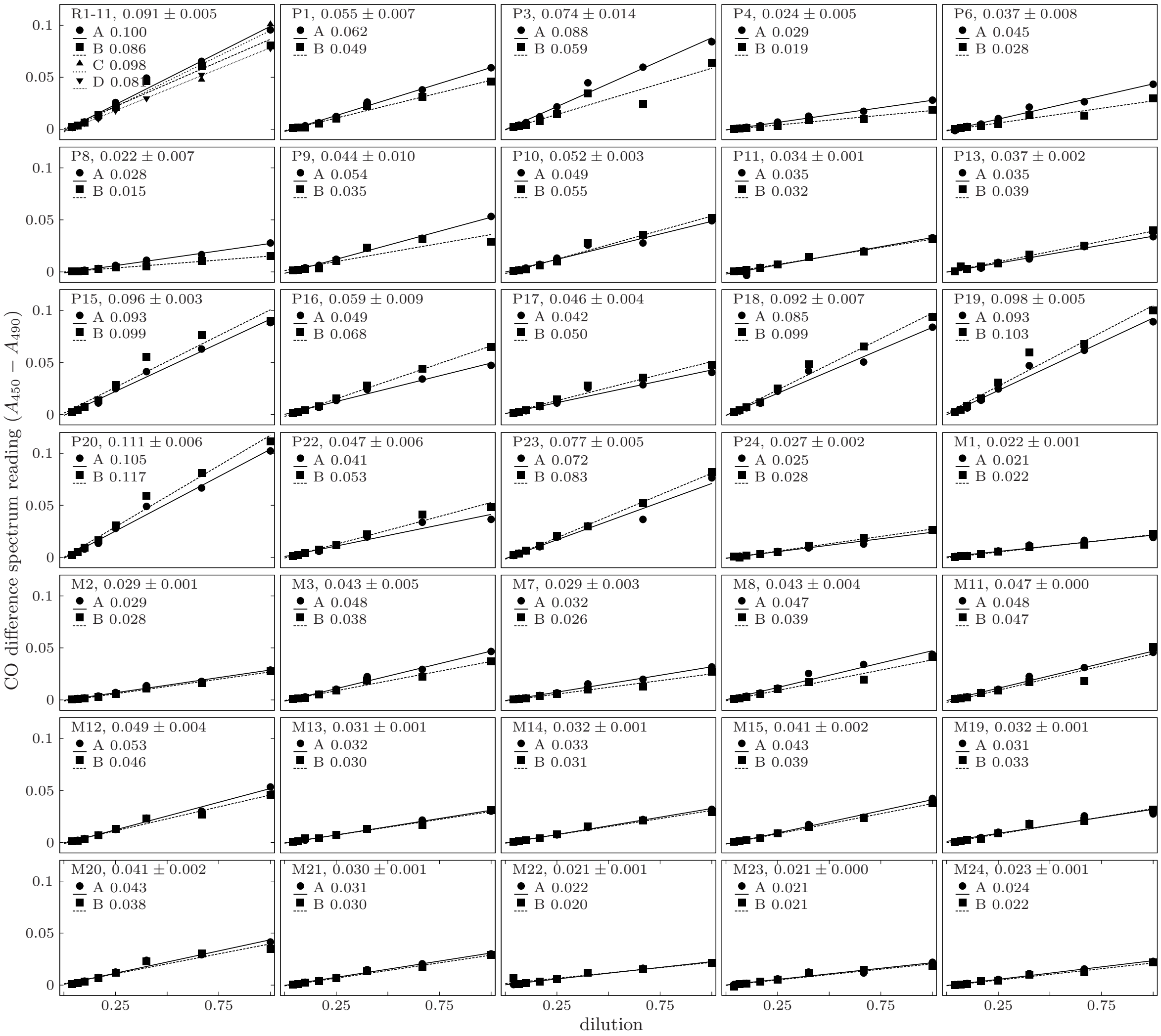

The standard curves used to determine the protein concentrations by CO difference spectrum measurements.

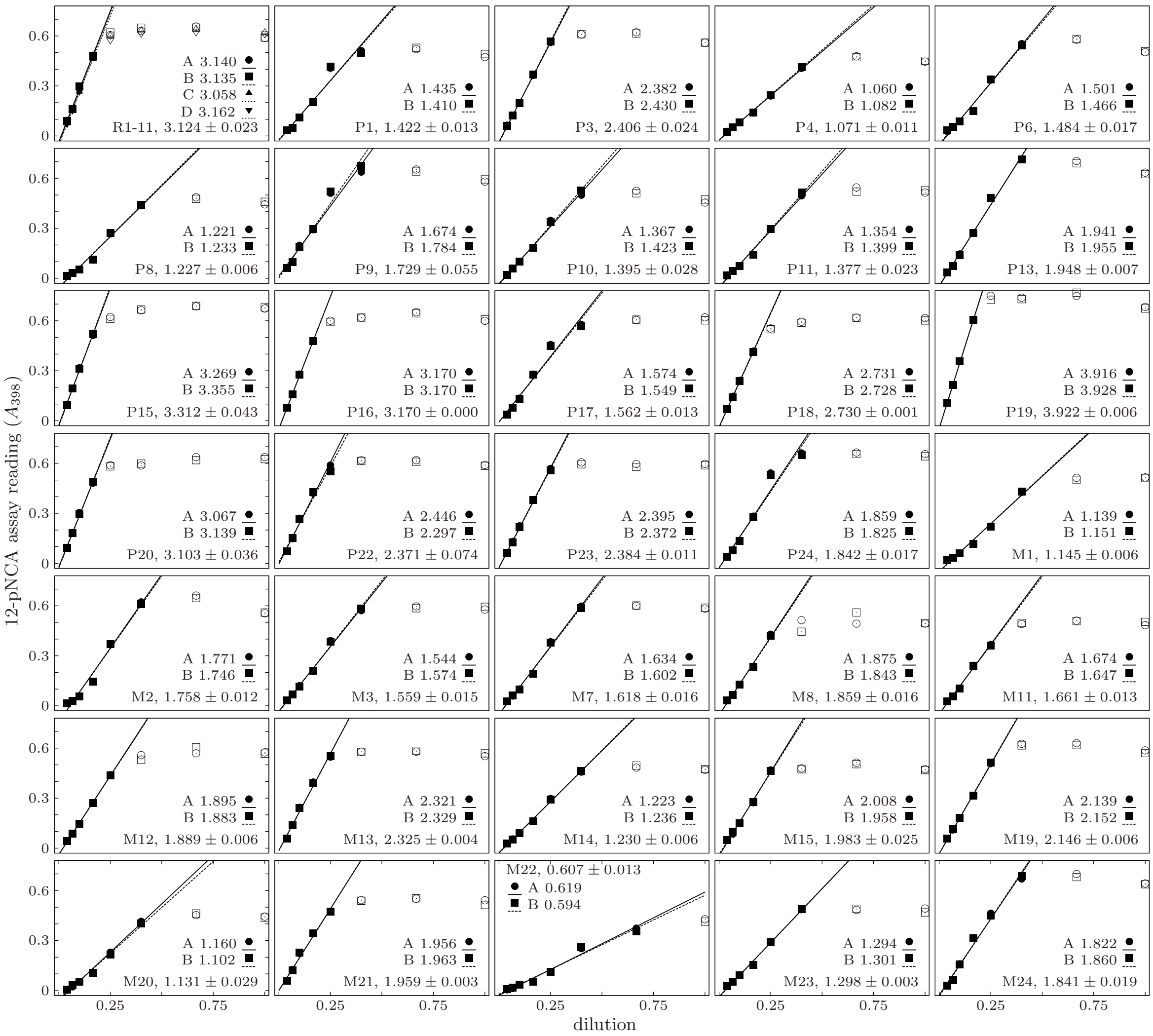

The standard curves used to determine the activities on 12-pNCA.

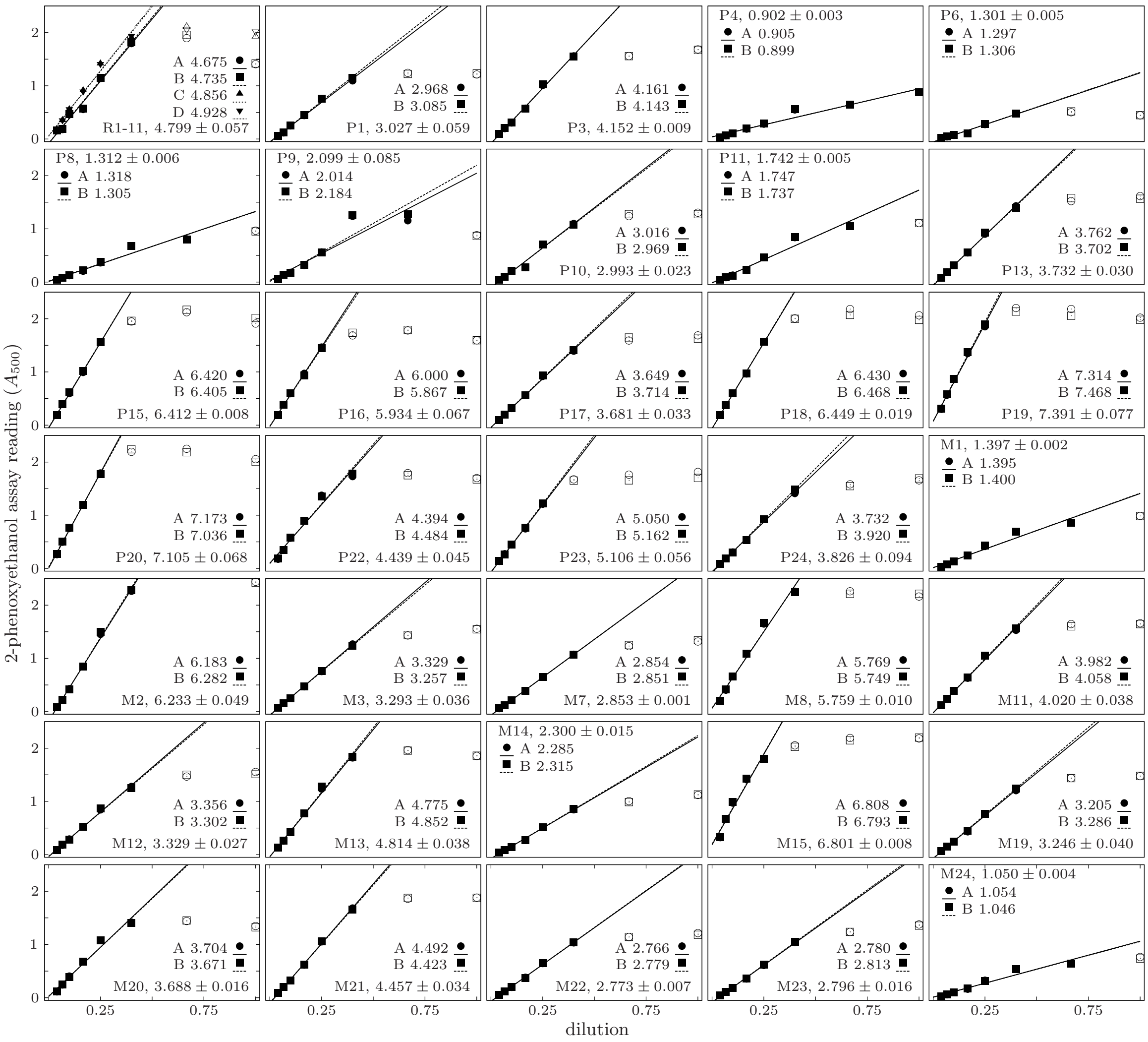

The standard curves used to determine the activities on 2-phenoxyethanol using the 4-AAP assay.

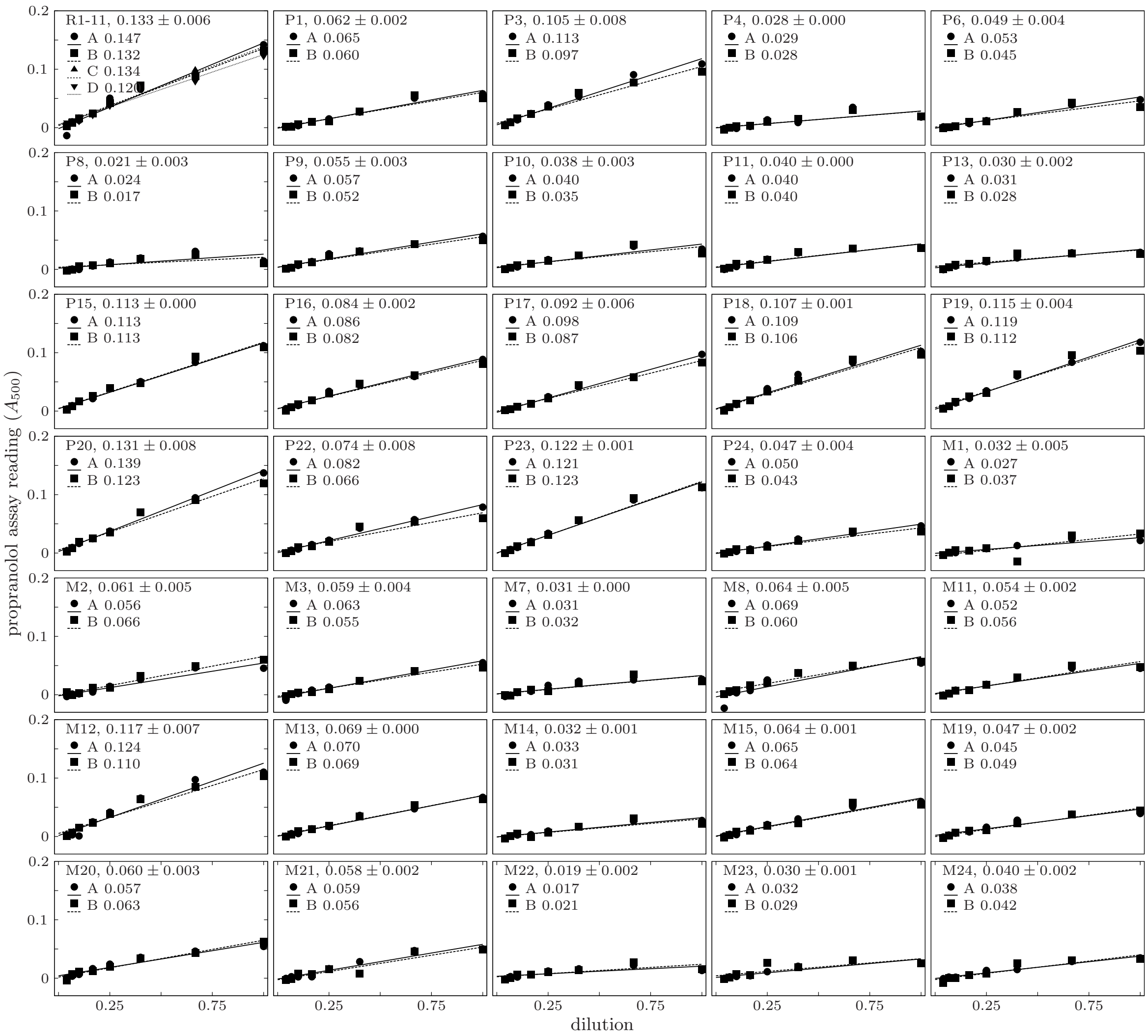

The standard curves used to determine the activities on propranolol using the 4-AAP assay.

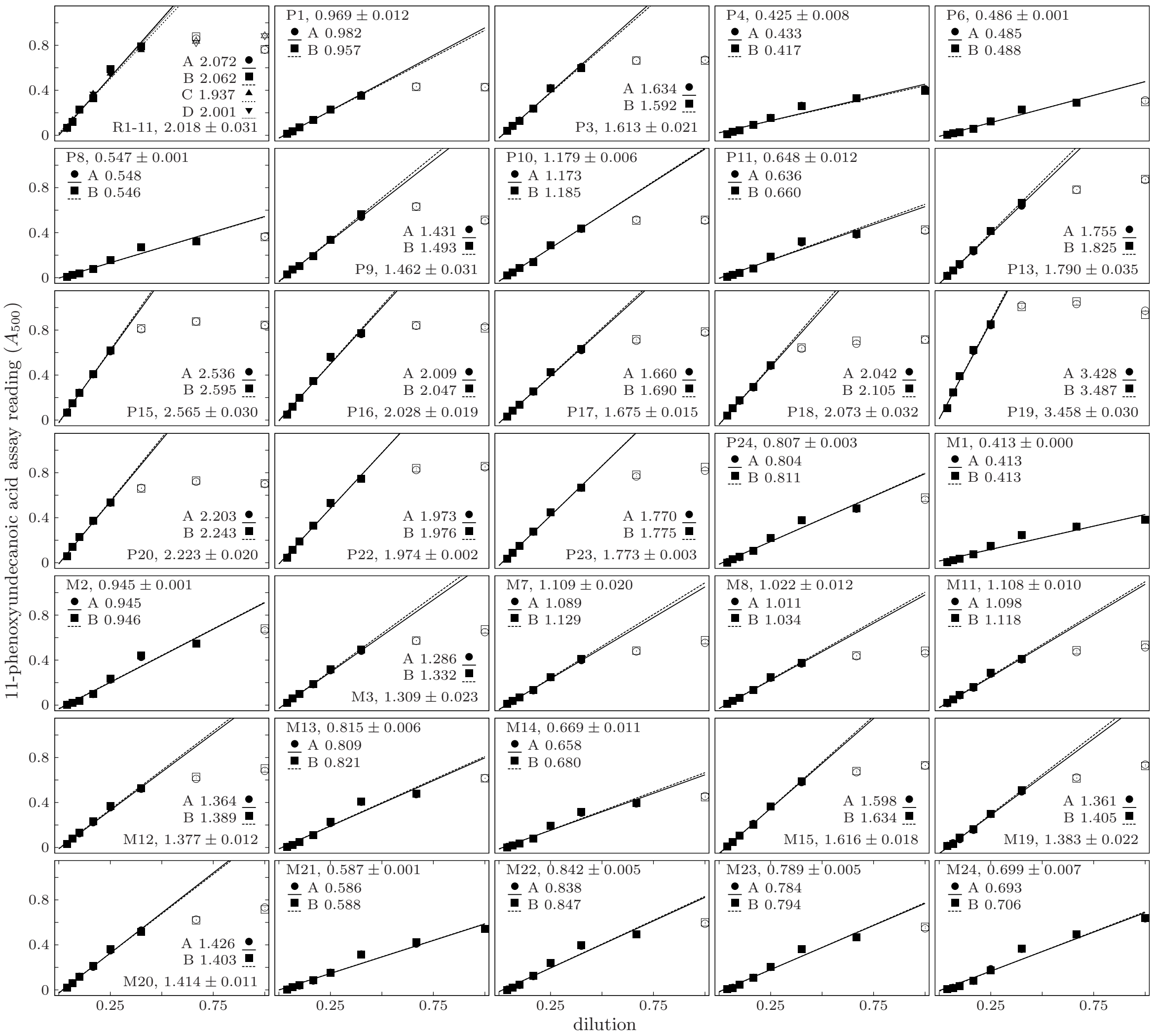

The standard curves used to determine the activities on 11-phenoxyundecanoic acid using the 4-AAP assay.

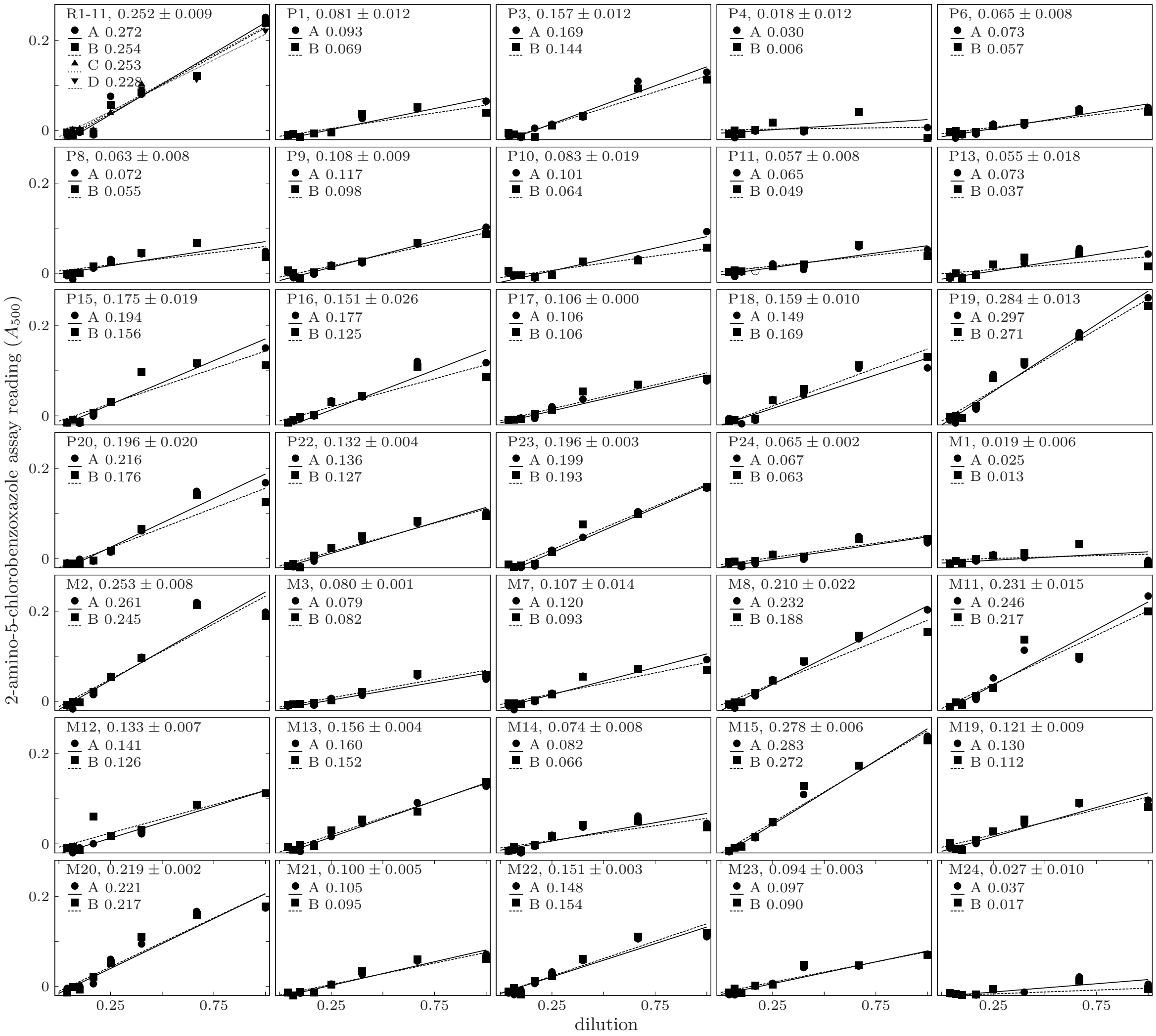

The standard curves used to determine the activities on 2-amino-5-chlorobenzoxazole using the 4-AAP assay.

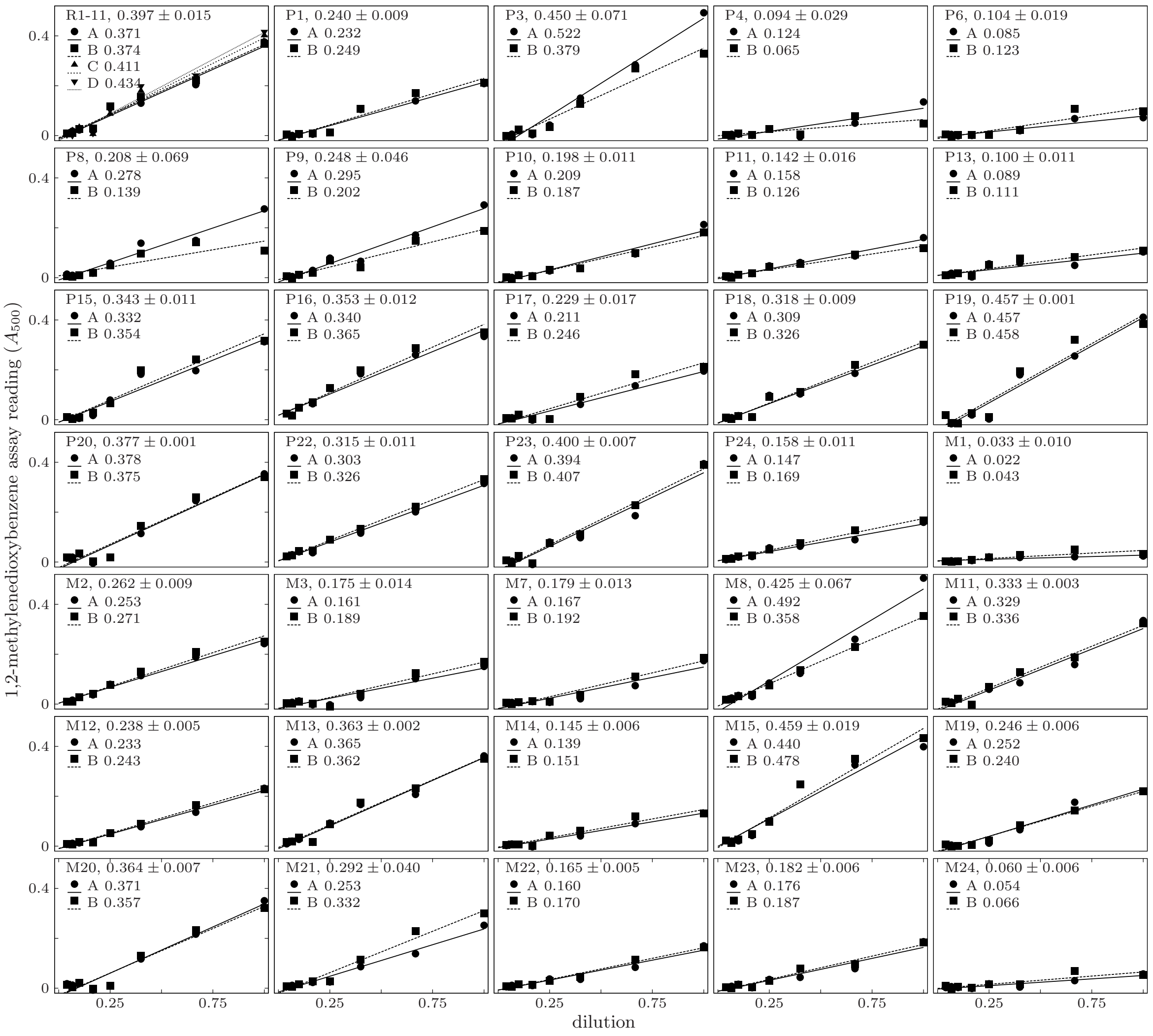

The standard curves used to determine the activities on 1,2-methylenedioxybenzene using the 4-AAP assay.
