# Supplementary material for: Neutral genetic drift can alter promiscuous protein functions, potentially aiding functional evolution
Source: Biol Direct. 2007 Jun 28;2:17. doi: 10.1186/1745-6150-2-17 (PMC1914045; doi:10.1186/1745-6150-2-17)
Supplement: Additional file 4 — Variability of experimental measurements. The attached PDF file shows the results of repeated activity measurements performed on the R1-11 parent P450. The plots are scaled so that they can be directly compared to Figure 3 to see how much of observed differences among neutrally evolved P450 variants might be due to variability in experimental measurements. Such a comparison indicates that the experimental measurement variability is much less than the differences between P450s. The plots also show that most of the experimental variation is introduced during the activity measurements rather than during expression/lysis. [file 1745-6150-2-17-S4.pdf]

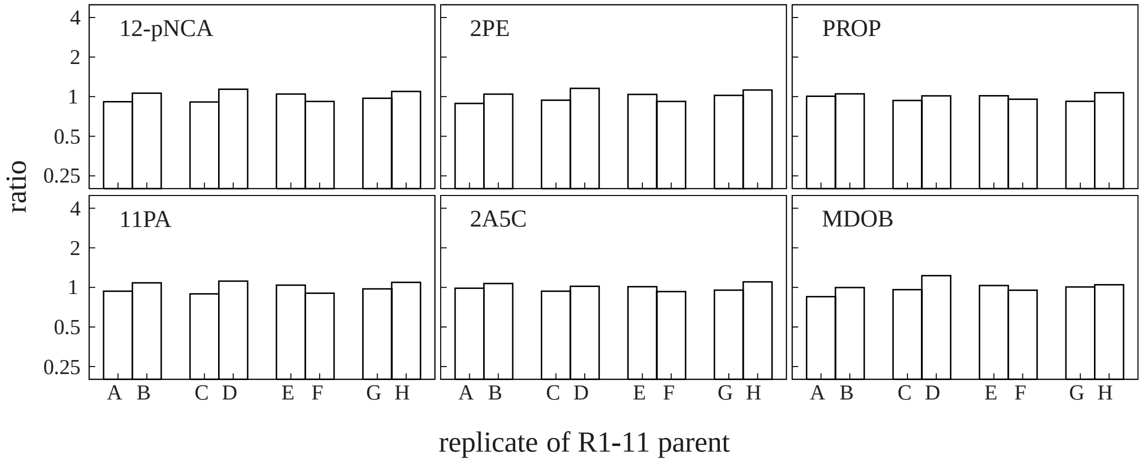

Additional file 4 - Variability of experimental measurements

The plots show that the readings from repeated activity measurements on different replicates of the R1-11 parent P450. Each letter indicates a different replicate. Replicates A and B are from the same expression culture and lysis preparation (with the protein concentration and activities measured independently), replicates C and D are from another expression/lysis preparation, and likewise for E/F and G/H. Replicates A, B, C, and D are the same as the ones with standard curves shown in Additional file 2, and all of the replicates are normalized to the same parental mean used in Figure 3. Therefore, these plots can be directly compared to Figure 3 to indicate how much of the differences in activity among the neutrally evolved P450 variants might be due simply to variation in the experimental measurements. As can be seen from such a comparison, the measurement variation is much less than the activity differences among most of the P450 variants. Comparison within and between the pairs of replicates from different expression/lysis preparations indicates that most of the experimental variability is introduced by the protein concentration and activity measurements rather than during the expression/lysis, since the difference within pairs is as large as the difference between pairs. This justifies making the duplicate measurements for the neutrally evolved P450 variants on samples from the same expression/lysis preparation.
